# Improved FAPI-radiopharmaceutical pharmacokinetics from the perspectives of a dose escalation study

**DOI:** 10.1007/s00259-025-07141-1

**Published:** 2025-02-26

**Authors:** Adrianna Bilinska, Sanjana Ballal, Chandrasekhar Bal, Tilman Läppchen, Eirinaios Pilatis, Elena Menéndez, Euy Sung Moon, Marcel Martin, Frank Rösch, Axel Rominger, Eleni Gourni

**Affiliations:** 1 Department of Nuclear Medicine, Inselspital, Bern University Hospital, Bern, Switzerland; 2 Department of Nuclear Medicine, All India Institute of Medical Sciences, New Delhi, India; 3 Department of Chemistry–TRIGA site, Johannes Gutenberg-University Mainz, Mainz, Germany

**Keywords:** FAPI, Circulating FAP, Improved pharmacokinetics, Gallium-68

## Abstract

**Purpose:**

This study explores the use of fibroblast activation protein inhibitors (FAPI) targeting radiopharmaceuticals as a new approach for pan-cancer treatment, focusing on key factors affecting their effectiveness. We hypothesized that adjusting the administered radiotracer dose one could enhance the tumor-to-background ratios.

**Methods:**

In a dose-escalation study with PC3 xenografts, all radiotracers were administered at doses between 10 and 1500 pmol, followed by biodistribution and PET/CT imaging. Their selectivity towards FAP, PREP, and DDP4, along with their stability in vivo, was assessed by biodistribution and metabolite analysis, respectively. Organ FAP expression was quantified using qPCR, and circulating FAP (sFAP) levels were measured in mouse and human blood samples via ELISA. Proof-of-principle human studies were also conducted.

**Results:**

Increasing the dose from 10 to 600 pmol significantly reduced blood uptake and enhanced tumor uptake, optimizing their in vivo performance. All radiotracers showed peak efficacy at 350–600 pmol, with altered pharmacokinetics beyond 600 pmol. Biodistribution studies validated the in vivo selectivity of all radiotracers towards FAP, even in the presence of PREP and DPP4 inhibitors, while they demonstrated remarkable stability in vivo. FAP expression was confirmed in various organs, with sFAP quantified in both healthy mice and humans. Human studies with [^68^Ga]Ga-DOTA.SA.FAPI revealed reduced off-target uptake (e.g., pancreas, salivary glands, heart), aligning with the preclinical findings.

**Conclusion:**

The study highlights the crucial need for precise FAPI-radiotracer dosing, optimizing PET imaging, reducing radiation exposure, and enhancing treatment by accounting for FAP biology and sFAP’s influence on pharmacokinetics.

**Supplementary Information:**

The online version contains supplementary material available at 10.1007/s00259-025-07141-1.

## Introduction

Fibroblast Activation Protein α (FAPα or FAP) is a cell-surface type II transmembrane glycoprotein overexpressed in the tumor microenvironment, specifically on the surface of cancer-associated fibroblasts (CAFs) and for some solid tumors on the tumor cell membrane as well. These expression profiles makes FAP an attractive target for cancer research, diagnosis and treatment, particularly in the development of theranostic radiopharmaceuticals [1, 2]. Despite their potential, FAPI-targeting radiopharmaceuticals face challenges in terms of high uptake in various organs, short tumor retention, and background uptake, mainly in blood [3–8]. The present study focuses on the rational to increase tumor uptake and tumor-to-background ratios.

**Fig. 1:**
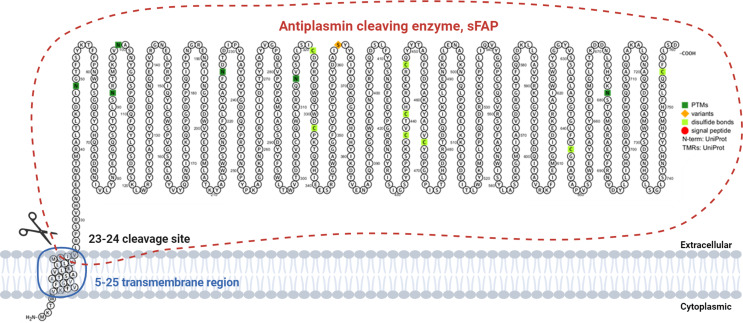
Schematic presentation depicting the human Fibroblast Activation Protein (FAP) and the Antiplasmin cleaving enzyme (soluble FAP) subsequent to FAP cleavage between amino acids 23–24. Amino acids 5–25 constitute a segment of the transmembrane region, which is not present in the soluble FAP (NP_032012.1) [10]. This visualization was generated using Protter to illustrate proteoforms (Omasits et al., Bioinformatics. 2013 Nov 21) and BioRender

Several factors may contribute to the background uptake: Firstly, a soluble form of FAP (sFAP) was found in both bovine and human plasma [9, 10], which is considered to be the product of protein shedding (Fig. 1). This phenomenon raises questions about how sFAP might influence the pharmacokinetics of radiopharmaceuticals designed to interact with FAP-positive tumors [11]. sFAP may compete with tumor-bound FAP for radiopharmaceutical binding, potentially affecting their intended targeting specificity, distribution and clearance kinetics [11, 12]. Furthermore, FAP undergoes alternative splicing, and multiple isoforms are expressed. However, the exact role of these isoforms remains unknown [12, 13]. Secondly, while FAP has traditionally been considered absent from adult tissues, more comprehensive FAP expression profiling in mice suggests that low basal levels of FAP expression might be found in various tissues, including uterus, bone marrow, adipose tissue, and pancreas [14]. Thirdly, the presence of other enzymes such as the dipeptidyl peptidase-4 (DPP4) and the prolyl oligopeptidase (PREP), which share exopeptidase and endopeptidase specificity with FAP respectively, might also influence the behavior of FAPI-radiopharmaceuticals [15–17].

These factors add significant complexity to the development and use of FAP-radiopharmaceuticals, making it essential to understand their interactions in order to develop strategies to optimize their in vivo performance.

In this comprehensive study, five FAPI-radiotracers, including two monomers (DOTA.SA.FAPI, DATA^5m^.SA.FAPI) and three dimers (DOTAGA.Glu.(FAPI)_2_, DO3A.Glu.(FAPI)_2_, DOTAGA.(SA.FAPI)_2_), (Fig. S1), all currently under clinical evaluation [3, 18–24], were systematically analyzed to address the aforementioned issues. Our investigation focused on: (i) quantifying FAP expression in healthy and PC3-mice as well as in blood; (ii) comparing these values with the uptake of the FAPI radiotracers in tumor, blood and organs; (iii) performing a dose escalation and in vivo selectivity investigation to assess the impact of sFAP, PREP and DPP-4 on the efficacy of the FAPI-radiotracers.

## Materials and methods

### Radiolabeling / quality control / lipophilicity

DOTA.SA.FAPI, DATA^5m^.SA.FAPI, DO3A.Glu.(FAPI)_2_, DOTAGA.(SA.FAPI)_2_ and DOTAGA.Glu.(FAPI)_2_ were labeled with gallium-68. The quality control of the ^68^Ga-labelled radiotracers was performed by reversed-phase high performance liquid chromatography (RP-HPLC) and radio thin layer chromatography (radio-TLC) (supplement).

Their lipophilicity (LogD_octanol/PBS_) was further determined (supplement).

### Cell lines / animal models

The study utilized the human prostate cancer cell line PC3 and cancer-associated fibroblast cell line CAF (hTERT PF179T CAF) from a male patient with prostate cancer. PC3 cells (3.5 × 10^6^/100 µL PBS) were implanted into the right shoulder of 6-week-old male athymic Balb/C nude mice (20–25 g; CByJ.Cg-Foxn1nu/J) to create tumor models for biodistribution and PET/CT studies at tumor sizes of 250–300 mm³. Healthy male athymic Balb/C mice were used for in vivo selectivity and RNA extraction (Animal License: BE98/2021).

### Metabolic stability

Metabolic studies were conducted 10 min after injecting 600 pmol (~ 4 MBq/100 µL) of each radiotracer in PC3-mice. Approximately 1 mL of blood was collected for plasma separation, and the stability of the ^68^Ga-labelled radiotracers was determined by RP-HPLC (supplement).

### Biodistribution study - dose escalation

PC3-mice were injected intravenously with 10-1500 pmol (0.03-4 MBq/100 µL) of all radiotracers. One hour p.i., the mice were euthanized for biodistribution studies (*n* = 3). For the 1000 and 1500 pmol doses, formulations included 600 pmol of each labeled radiotracer with 400 or 900 pmol of the respective non-labeled precursor (supplement).

### Small-animal PET/CT imaging

PET/CT scans were acquired using a dedicated micro-PET/SPECT/CT scanner (Albira Si; Bruker Biospin, Ettlingen, Germany). Static PET images were obtained 1 h after injection of 100–1500 pmol (0.3–4 MBq / 100 uL) of [^68^Ga]Ga**-**DOTAGA.Glu.(FAPI)_2_, [^68^Ga]Ga**-**DO3A.Glu.(FAPI)_2_, [^68^Ga]Ga**-**DOTAGA.(SA.FAPI)_2_, [^68^Ga]Ga**-**DOTA.SA.FAPI and [^68^Ga]Ga**-**DATA^5m^.SA.FAPI on a PC3 xenograft mouse model (*n* = 3/group). The mice were scanned for 20–30 min, under 2% isoflurane anesthesia as a mixture of O_2_ with a 0.8 LPM flow. (supplement).

### RNA extraction

Organs of interest of healthy (*n* = 2) and PC3-mice (*n* = 2) were collected to an RNAlater^®^ solution. Total RNA extracted from organs and blood. The RNA concentrations and quality were determined using a NanoDrop 2000 spectrophotometer (supplement).

### Real time PCR

One microgram of RNA was reversely transcribed using a High-Capacity cDNA kit. The design of the primer sets for quantitative real-time PCR and the assessment of relative transcript levels are given in the supplement. Standard curves were designed as tenfold dilutions of the PCR products. Relative transcript levels of all samples were calculated after normalization with the transcript level of reference genes of GAPDH and RLPL0.

### ELISA

Approximately 1 mL of blood was collected from PC3-mice (*n* = 6), healthy mice (*n* = 4) and healthy human volunteers (*n* = 4), followed by plasma separation. Cell culture media from CAFs was collected after 10 days of culture in T75 flask without media change. ELISA was performed using plasma and cell culture media samples following the manufacturer protocol.

### In vivo selectivity of the Radiolabeled Tracers towards FAP

Competition experiments were performed in 8 cohorts of healthy mice. Ten pmol of [^68^Ga]Ga-DOTAGA.Glu.(FAPI)_2_ (0.08–0.09 MBq/100 µL) was co-injected intravenously with varying combinations of 500 pmol of DOTAGA.Glu.(FAPI)_2_, 3 different PREP (Salidroside Baicalin, KYP-2047) and a DPP4 (DPP IV) inhibitor. The PREP and DPP4 inhibitors were commercially available: DPP4 inhibitor (Sigma Aldrich, Millipore, Germany), Baicalin (Sigma Aldrich, Germany), Salidroside (Lubio Science, Switzerland), KYP-2047 (Sigma Aldrich, Germany). All inhbitors were diluted to final concentration 500 pmol following manufacturer leads. Biodistribution studies were conducted 1 h p.i.

In a separate group of PC3 tumor bearing mice, the injected mass of [^68^Ga]Ga-DOTAGA.Glu.(FAPI)_2_ which led to the highest tumor uptake, 600 pmol (~ 4 MBq/100 µL), was co-injected intravenously with 500 pmol of each of the DPP4 and PREP (Salidroside) inhibitors. Biodistribution studies were conducted 1 h p.i. All animals were terminally anesthetized by intraperitoneal injection of an overdose of pentobarbital natrium (150 mg/kg; Streuli Pharma SA) at 1 h after injection of the radiotracers. The organs of interest were dissected and weighted, and the radioactivity in tissue samples was counted in a γ-counter. Biodistribution data are given as percent of injected activity per gram of tissue (% IA/g) and are means ± SD (*n* = 3).

### Comparison of non-target organ uptake between lower and higher injected masses of ^68^Ga-DOTA.SA.FAPI in PET imaging in clinical studies

To evaluate the impact of the injected mass on the pharmacokinetics of [^68^Ga]Ga-DOTA.SA.FAPI in patients, two different total injected masses - low and high - of [^68^Ga]Ga-DOTA.SA.FAPI were administered to four patients in a head-to-head comparison. Each patient received both dosages over a span of approximately two years. Initial [^68^Ga]Ga-DOTA.SA.FAPI PET/CT was performed 1 h p.i. with a lower injected mass (7.8–11 µg) and activity levels of 81.4–133.2 MBq. Within a post-treatment follow-up, another [⁶⁸Ga]Ga-DOTA.SA.FAPI PET/CT was performed using a higher injected mass (40–50 µg) with activity levels of 111–122 MBq, 1 h p.i.

### Statistical analysis

A two-way ANOVA was performed to assess the significance of biosdistribution, FAP transcript data and clinical data (supplement). All analyses were done in Prism 8 to determine statistical significance at the 95% confidence level, with a P-value < 0.05 considered significant.

## Results

### Radiolabeling / quality control / lipophilicity

The FAPI-ligands were successfully labelled with gallium-68 in > 98% radiochemical purity (Fig. S3-8), with apparent molar activities (A_m_) ranging from 9 to 22 GBq/µmol. No colloids formed. The LogD_octanol/PBS−pH7.4_ values for [^68^Ga]Ga-DOTA.SA.FAPI, [^68^Ga]Ga-DATA^5m^.SA.FAPI were − 3.4 ± 0.03 [3] and − 3.6 ± 0.1 [4], respectively. The LogD_octanol/PBS−pH7.4_ values for [^68^Ga]Ga-DOTAGA.Glu.(FAPI)_2,_ [^68^Ga]Ga**-**DO3A.Glu.(FAPI)_2_ and [^68^Ga]Ga**-**DOTAGA.(SA.FAPI)_2,_ were found to be -2.9 ± 0.1, -2.2 ± 0.04 and − 1.8 ± 0.02 [3], respectively.

### Metabolic stability

**Fig. 2:**
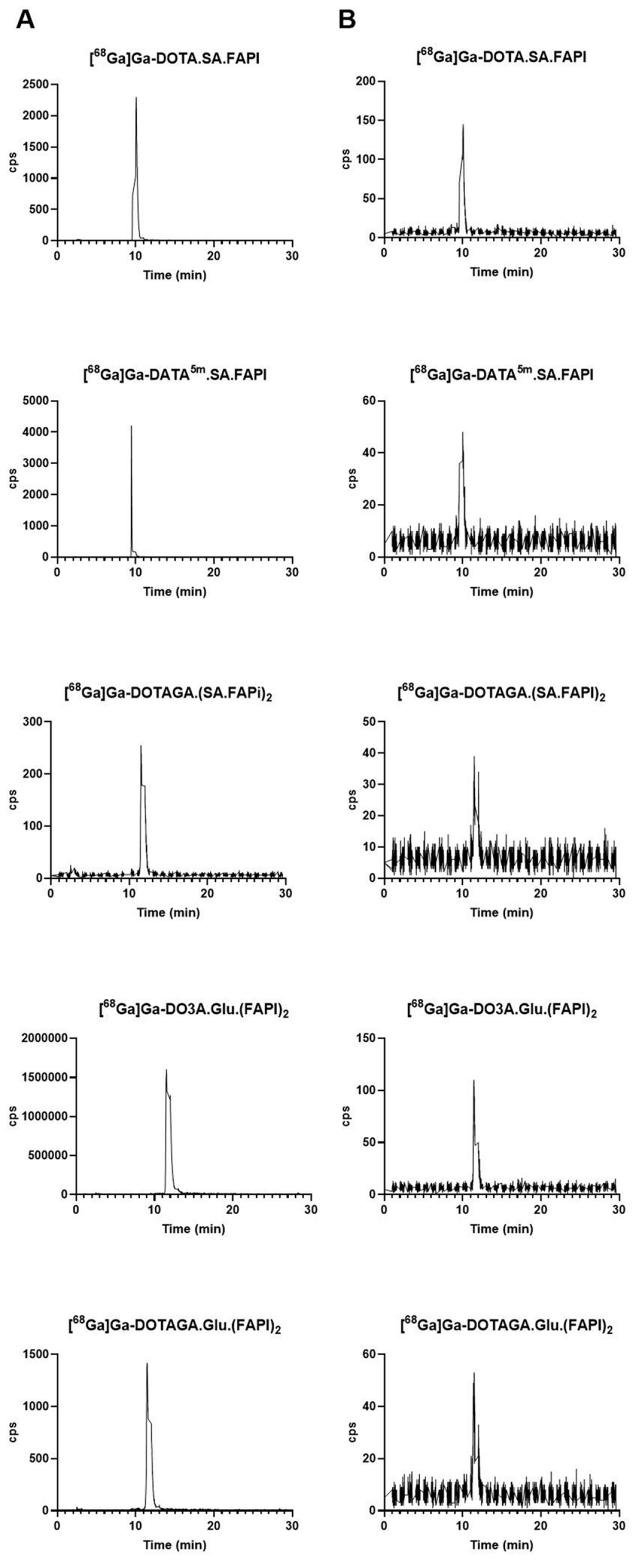
Representative radio-HPLC chromatograms of the radiotracers are shown in two panels. Panel (**A**) presents reference chromatograms for the radiotracers before their injection into PC3-mice. Panel (**B**) displays the chromatograms from blood samples collected 10 min p.i. of the respective radiotracers

The HPLC analysis of plasma samples (Fig. 2) demonstrates that the circulating radioactivity in the blood predominantly consists only intact radiotracer in each case.

### Biodistribution study / dose escalation

**Fig. 3:**
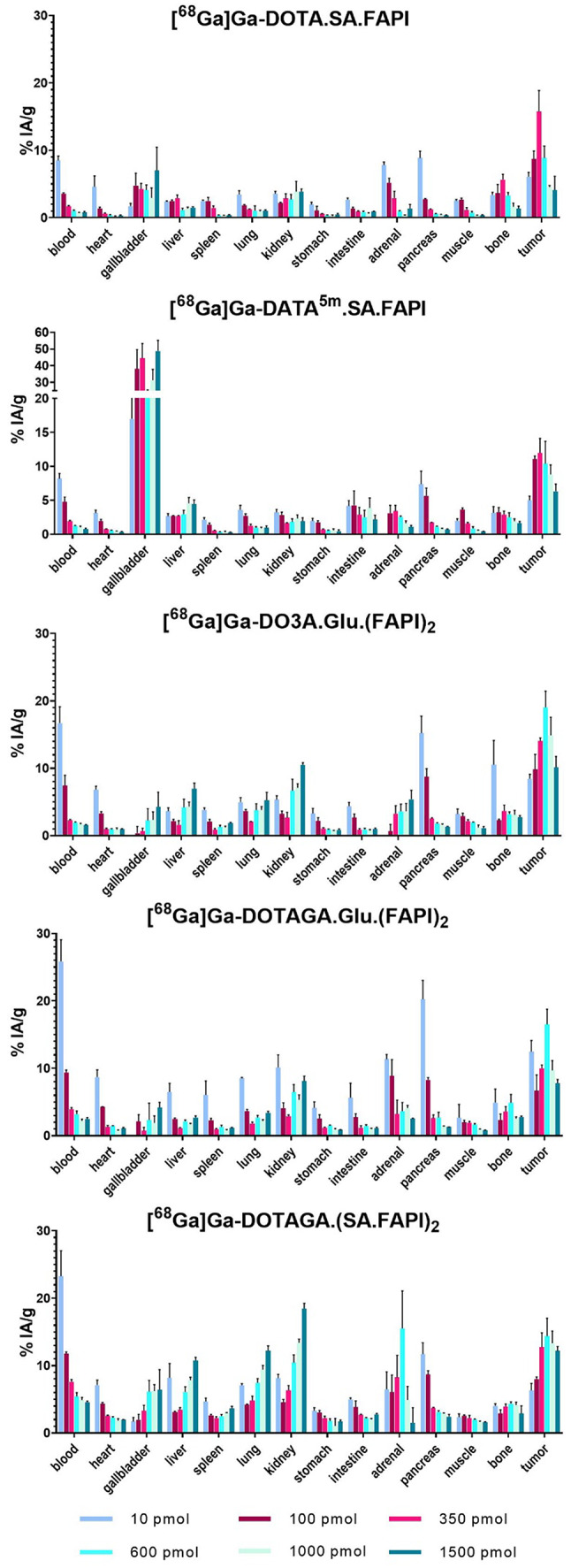
Biodistribution data of the ^68^Ga-labeled radiotracers in PC3-mice at 1 h p.i. Data have been calculated as %I.A./g of tissue and are presented as mean ± SD (*n* = 3)

**Fig. 4:**
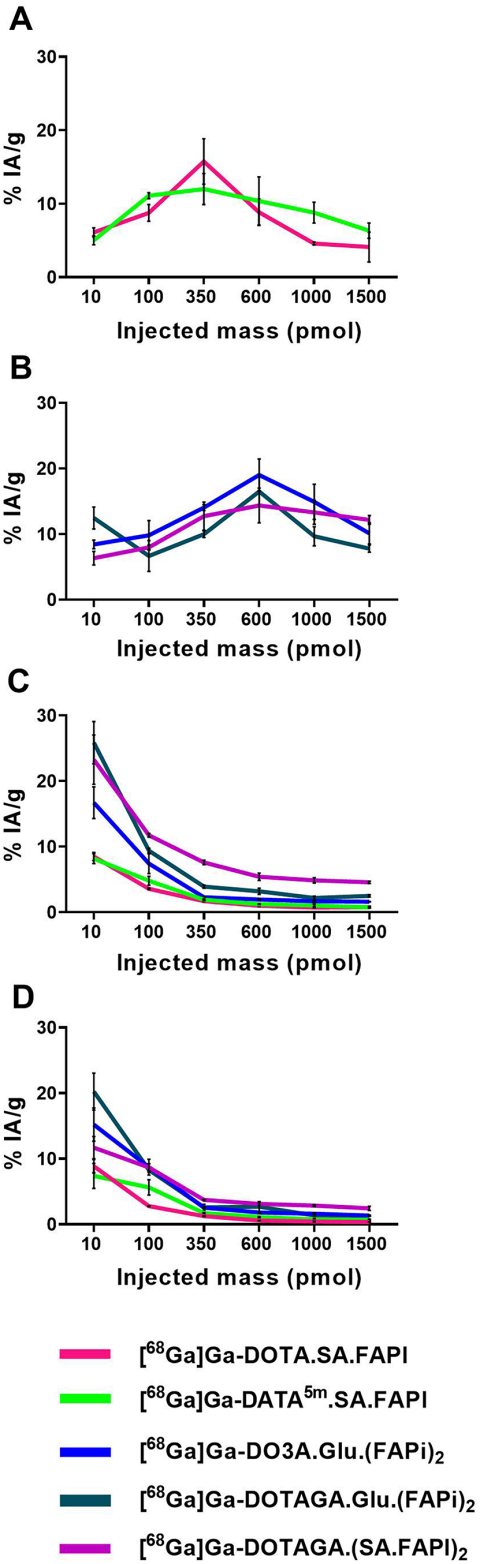
Tumor (**A** and **B**), blood (**C**) and pancreas (**D**) uptake of the ^68^Ga-labeled radiotracers in PC3-mice at 1 h p.i. at injected amounts of 10, 100, 350, 600, 1000 and 1500 pmol of the respective radiotracers

The impact of FAPI-radiotracers mass in PC3-mice was evaluated through a dose escalation study, (Figs. 3 and 4, Tables S1-S5). The highest tumor uptake (12-19% IA/g) occured at doses of 350–600 pmol. Administering 1000 pmol caused a marked reduction in tumor uptake that decreases by factors of 0.3, 0.7, 0.9, 0.6, and 0.8 for [^68^Ga]Ga-DOTA.SA.FAPI, [^68^Ga]Ga-DATA^5m^.SA.FAPI, [^68^Ga]Ga-DO3A.Glu.(FAPI)_2_, [^68^Ga]Ga-DOTAGA.Glu.(FAPI)_2_, and [^68^Ga]Ga-DOTAGA.(SA.FAPI)_2_, respectively, relative to their maximum uptake. A further increase to 1500 pmol led to an additional reduction.

**Fig. 5:**
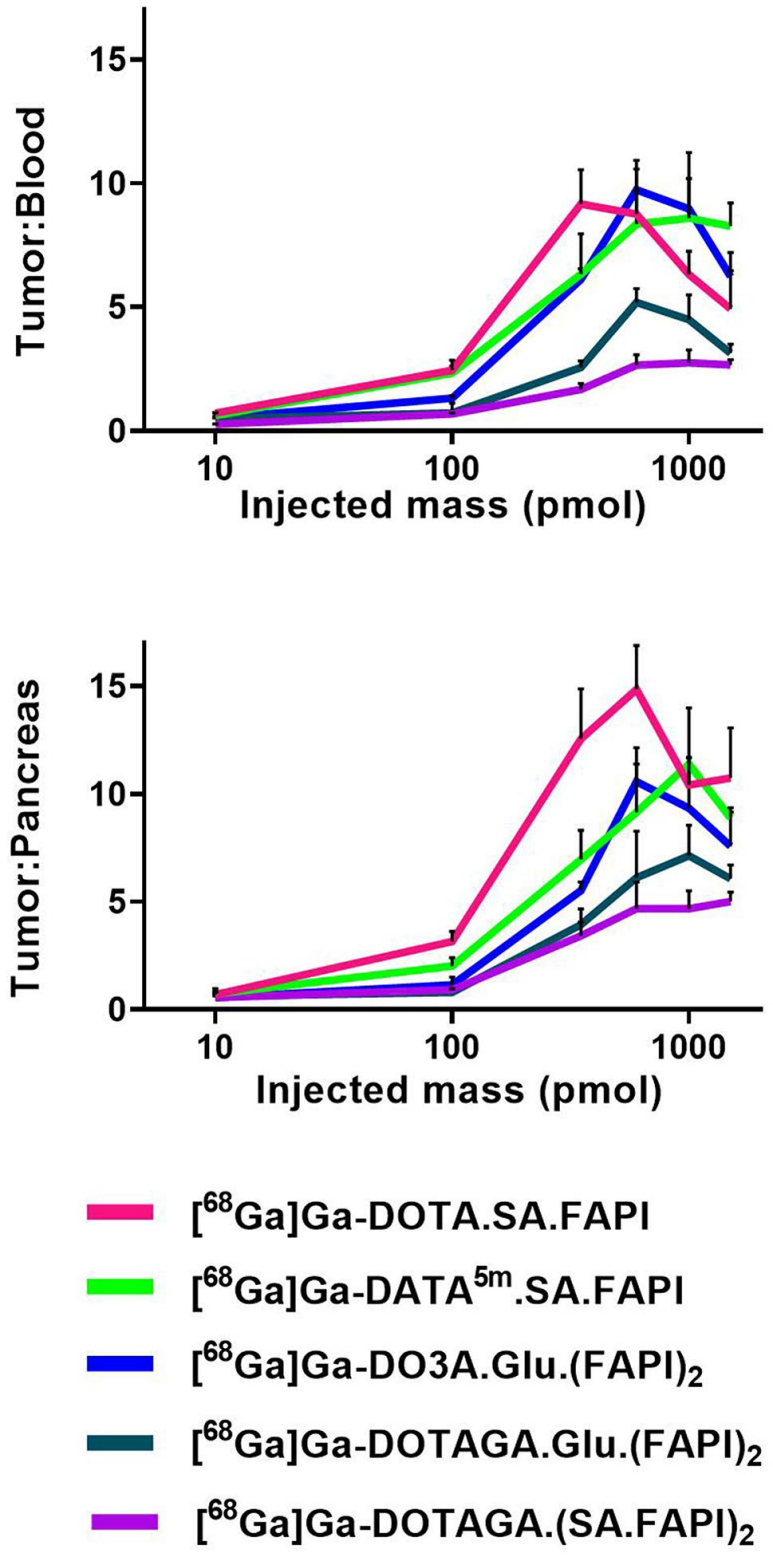
Tumor-to-blood and tumor-to-pancreas activity concentration ratios of the respective radiotracers (x-axis is presented on a logarithmic scale)

The increasing radiotracer mass led to a significant reduction in blood uptake. At 10 pmol, blood uptake was the highest (~ 9% IA/g for monomers and ~ 20% IA/g for dimers), decreasing to ~ 1% IA/g for monomers and 2–5% IA/g for dimers at 600–1500 pmol. The tumor-to- blood and pancreas ratios (Fig. 5) demonstrate a clear advantage of the monomers compared to the dimers in achieving higher contrast, given an equal administered cold mass.

### Small-animal PET/CT studies

**Fig. 6:**
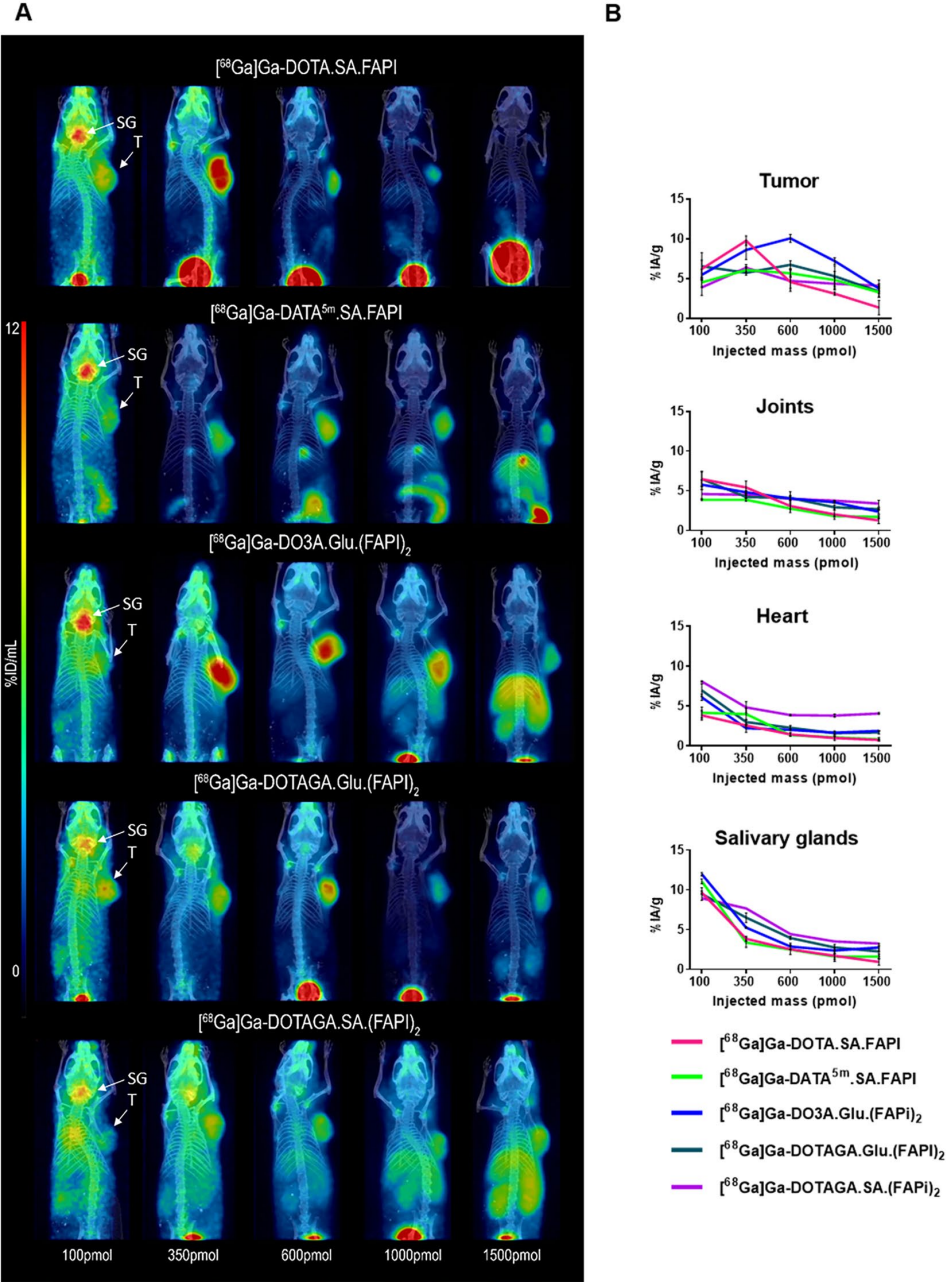
(**A**) PET imaging of the ^68^Ga-labeled radiotracers in PC3-mice at 1 h p.i at injected amounts of 10, 100, 350, 600, 1000 and 1500 pmol of the respective radiotracers. T- tumor, SG- salivary glands. (**B**) Quantitative analysis of selected organs (tumor, joints, heart and salivary glands) of the PET images

PET/CT imaging (Fig. 6) aligns closely with the biodistribution data, clearly indicating the optimal injected mass for maximum tumor uptake per compound. PET data highlight the areas where blood uptake decreases and demonstrate the changes in pharmacokinetics observed when the injected mass exceeds 1000 pmol.

### Real time PCR / ELISA

**Fig. 7:**
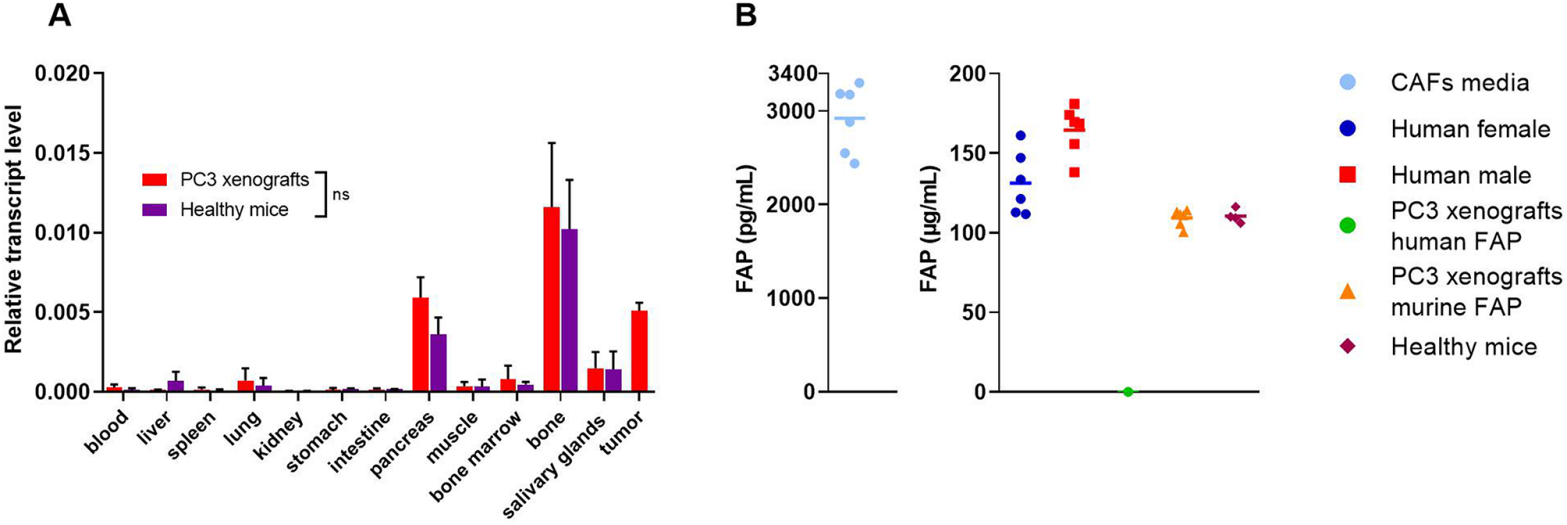
(**A**) Relative mRNA levels of FAP in selective organs obtained from PC3-mice and healthy mice. The levels of FAP transcripts in two groups showed no significant differences (*p* < 0.05). (**B**) Concentration of human and murine FAP in CAFs culture media, healthy human and murine plasma

Quantitative PCR showed the highest FAP transcript levels in bone, tumor, pancreas, salivary glands and bone marrow in both PC3 and healthy mice (Fig. 7A). These organs exhibited increased radioactive uptake and displayed a pattern of blocking when the administered dose of the radiotracers exceeded 100 pmol (Fig. 3). Human FAP levels in CAF culture media were 6 orders of magnitude lower compared to mouse and human plasma. Murine sFAP levels were comparable in plasma sample of PC3 and healthy mice while human sFAP was not detected. The presence of sFAP was also confirmed in human plasma (Fig. 7B).

### In vivo selectivity of the radiolabeled tracers towards FAP

**Fig. 8:**
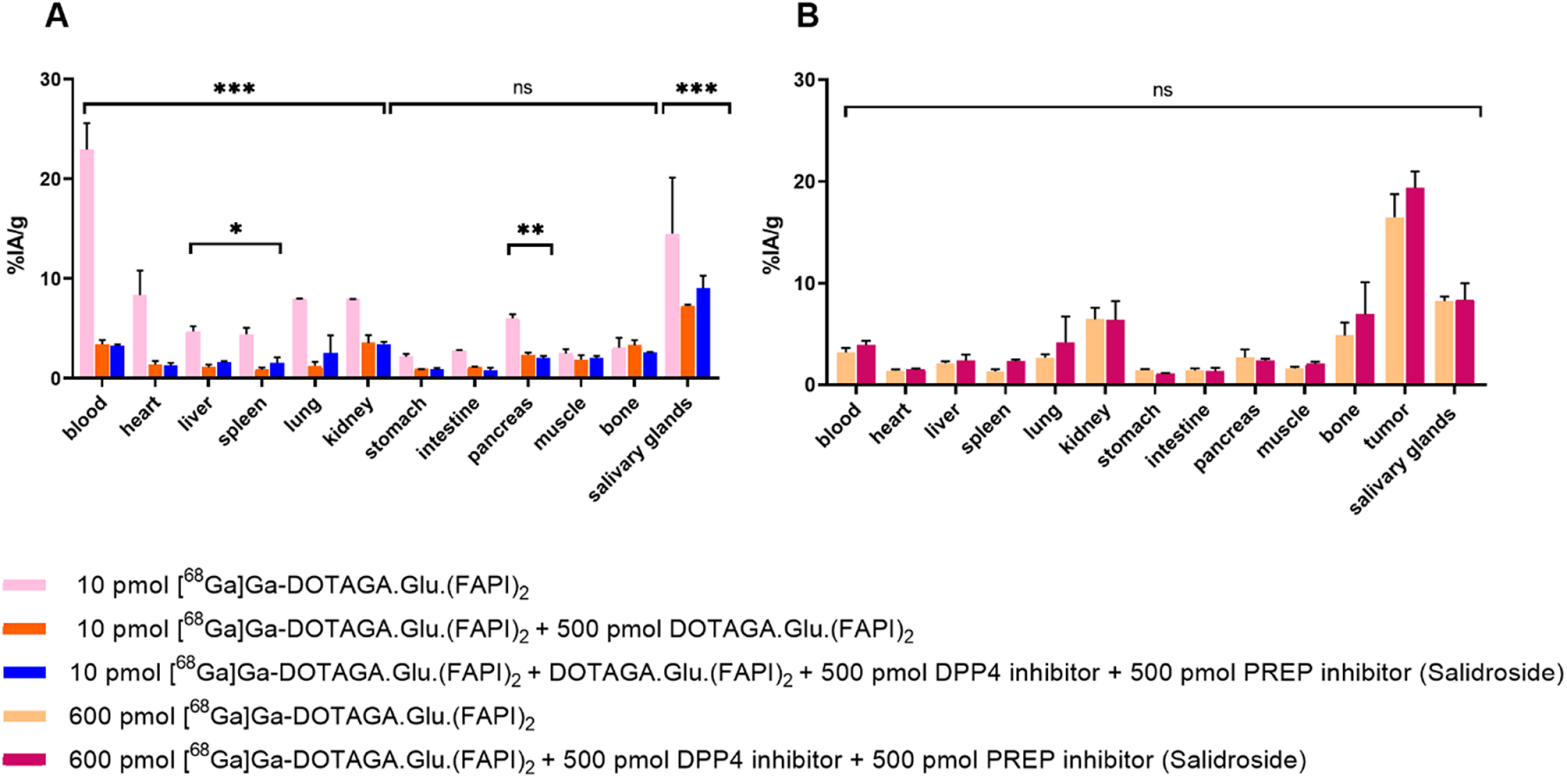
Biodistribution data (**A**) from healthy mice injected with 10 pmol [^68^Ga]Ga-DOTAGA.Glu.(FAPI)_2_ alone, co-injected with 500 pmol DOTAGA.Glu.(FAPI)_2_, or co-injected with 500 pmol DOTAGA.Glu.(FAPI)_2_, 500 pmol PREP (Salidroside), and 500 pmol DPP4 inhibitor. (**B**) From PC3-mice receiving 600 pmol [^68^Ga]Ga-DOTAGA.Glu.(FAPI)_2_, either alone or co-injected with 500 pmol PREP (Salidroside) and 500 pmol DPP4 inhibitor. Statistical significance was determined by comparison to control groups (**A**: 10 pmol [^68^Ga]Ga-DOTAGA.Glu.(FAPI)_2_; **B**: 600 pmol [^68^Ga]Ga-DOTAGA.Glu.(FAPI)_2_) with significance levels: *p* < 0.001(***), 0.002 (**), 0.033 (*), 0.12 (ns)

To evaluate the selectivity of the radiotracers towards FAP, the injected mass of 10 pmol of [^68^Ga]Ga-DOTAGA.Glu.(FAPI)_2_ served as reference and was further co-injected with various combinations of the DOTAGA.Glu.(FAPI)_2_, PREP, and DPP4 inhibitors (Fig. 8A, Fig. S9, Tables S6,S7). Co-injection of [^68^Ga]Ga-DOTAGA.Glu.(FAPI)_2_ with the PREP- and DPP4-inhibitors, either alone or in several combinations, did not lead to reduced background uptake. In contrast, co-injection with 500 pmol DOTAGA.Glu.(FAPI)_2_ resulted in reduced background uptake in all organs, both alone and in all combinations with the PREP- and DPP4-inhibitors. The only significant change was observed in the pancreas, where co-injection generally led to an increased pancreatic uptake (Table S7). Remarkably, the presence of DOTAGA.Glu.(FAPI)_2_ in blood circulation reduced blood uptake from about 23% IA/g to approximately 3% IA/g. Uptake in other organs was also reduced when a cold mass (500 pmol) of DOTAGA.Glu.(FAPI)_2_ was co-injected with [^68^Ga]Ga-DOTAGA.Glu.(FAPI)_2_.

Co-injecting 500 pmol of each of DDP4 and PREP inhibitors along with 600 pmol of [^68^Ga]Ga-DOTAGA.Glu.(FAPI)_2_, (Fig. 8B, Table S8), did not affect either tumor uptake or the general pharmacokinetics compared to the biosdistribution data obtained after injecting 600 pmol of [^68^Ga]Ga-DOTAGA.Glu.(FAPI)_2_.

### Comparison of non-target organ uptake between lower and higher injected masses of ^68^Ga-DOTA.SA.FAPI in PET imaging in clinical studies

**Fig. 9:**
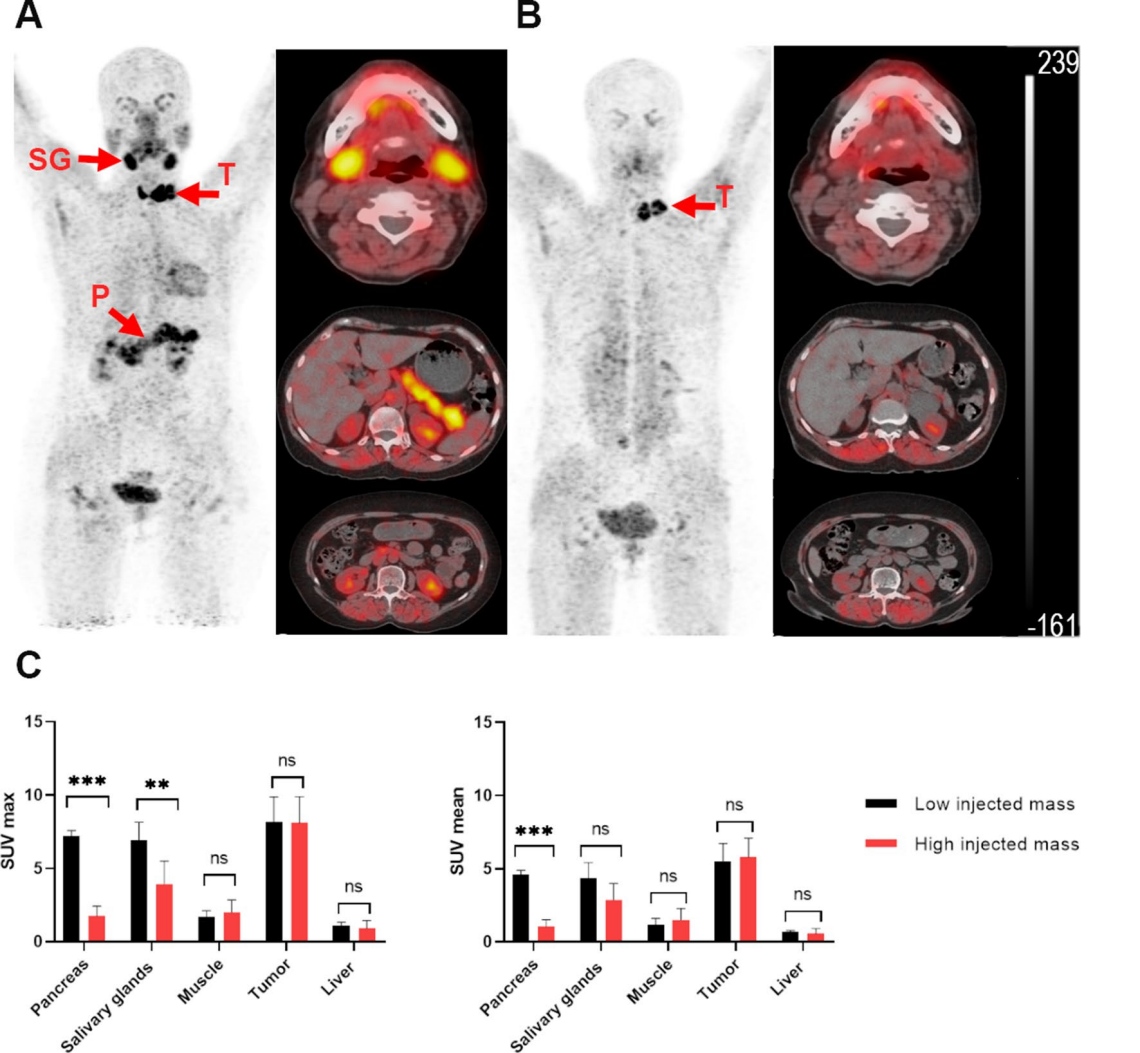
[^68^Ga]Ga-DOTA.SA.FAPI PET/CT images of a representative 60 year-old female patient with papillary thyroid cancer. The patient was injected in total with 7.6 µg (**A**) and 50 µg (**B**) of [^68^Ga]Ga-DOTA.SA.FAPI. (**C**). Quantitative analysis of the PET images (SUVmax and SUVmean) for pancreas, salivary glands, muscle, liver parenchyma and tumor lesions. Statistical significance was determined by comparison of low and high injected mass of [^68^Ga]Ga-DOTA.SA.FAPI (for SUVmax and SUVmean) with significance levels: *p* < 0.001(***), 0.002 (**), 0.033 (*), 0.12 (ns)

PET/CT scans performed with lower masses of [^68^Ga]Ga-DOTA.SA.FAPI (Fig. 9A) typically exhibited high uptake in both lesions and FAP-expressing healthy organs. In contrast, imaging using higher masses of [^68^Ga]Ga-DOTA.SA.FAPI revealed markedly reduced uptake in non-target organs (Fig. 9B). SUV max values for the pancreas and salivary glands significantly decreased with higher doses injected (pancreas: *p* < 0.001; salivary glands: *p* < 0.003) (Table 1; Fig. 9C). SUV mean values for the pancreas (*p* < 0.001) significantly decreased with higher doses injected, while the salivary glands did not exhibit significant changes; however, the p-value of 0.06 suggests that the results are close to achieving statistical. Notably, uptake in the liver parenchyma and muscle remained consistent across both dose levels (*p* > 0.99). Importantly, lesion uptake showed no significant difference between low and high masses of [^68^Ga]Ga-DOTA.SA.FAPI (*p* > 0.99).

**Table 1:** SUV max and SUV mean of various regions after the injection of low (A) and high (B) mass of [^68^Ga]Ga-DOTA.SA.FAPI

	A	B	A	B
Organ	SUV max Median (IQR)		SUV mean Median (IQR)	
Pancreas	7.2 (6.8–7.6)	1.7 (1.1–2.5)	4.6 (4.3–4.9)	1.0 (0.7–1.6)
Salivary glands	7.2 (5.1–7.7)	3.7 (2.6–5.8)	4.3 (3.3–5.5)	2.7 (1.9–4.2)
Liver	1.1 (0.8–1.4)	0.7 (0.5–1.7)	0.7 (0.6–0.8)	0.4 (0.3–1.1)
Muscle	1.6 (1.3–2.2)	2.0 (1.2–2.9)	1.2(0.8–1.6)	1.6 (0.6–2.2)
Tumour/Lesion	8.4 (5.9–10.0)	7.9 (6.1–10.5)	5.6 (4.0-6.9)	5.7 (4.3–7.5)

## Discussion

FAPI-radiopharmaceuticals have shown great promise in targeting FAP across preclinical and clinical studies [3, 5–8, 16, 18–23, 25–34]. A consideration in their clinical use is the blood uptake, observed especially in the case of the dimers mainly at early time points after their injection. This may affect the distribution of the dose between circulation and tumor site [4–7, 26, 28, 33–35]. For PET imaging, the blood signal lowers the contrast while in therapies it reduces the dose available for tumor targeting. Addressing these issues necessitates not only optimizing the design of FAPI-ligands but also adjusting the formulation of the injectable FAPI-radiopharmaceuticals to optimize their pharmacokinetics and minimize non-specific uptake. This work focused on the later aspect.

FAP exists in multiple isoforms, which arise from alternative splicing or post-translational modifications [10, 12]. One such isoform is sFAP that lacks the transmembrane domain (Fig. 1), allowing it to be secreted into the extracellular space and circulate in blood [10]. This study primarily aims to investigate the extent to which sFAP competes with tumor-bound FAP and its impact on the pharmacokinetics of FAPI-radiopharmaceuticals.

The dose-escalation study of five FAPI-radiotracers in PC3-mice demonstrated a dose-dependent increase in tumor uptake, as evidenced by both biodistribution and PET studies. Specifically, an injected mass of radiotracers ranging from 350 to 600 pmol resulted in maximum accumulation of the radioactivity in the tumors (Figs. 3 and 4). When the injected doses were increased up to 1500 pmol, there was a significant reduction in tumor uptake and substantial alteration of their pharmacokinetic behavior. This reduced tumor uptake is most likely attributable to receptor saturation by the increased mass of the unlabeled compound in the circulation. In addition, FAP-positive organs, including salivary glands, pancreas, bones and blood, as confirmed by qPCR, exhibited a pattern of blocking when the administered dose exceeded 100 pmol, thus, explaining the unspecific binding and high background uptake at low injected masses (Figs. 3 and 7A). Moreover, FAP relative mRNA levels in the pancreas were comparable between healthy and PC3-mice. Nevertheless, biodistribution data indicated a significant difference in pancreatic uptake of [^68^Ga]Ga-DOTAGA.Glu(FAPI)_2_ between these two groups. This suggests possible alterations at the protein level that may be influencing tracer binding and uptake, independent of mRNA expression.

Galbiati et al. recently investigated how molar dose affects the in vivo tissue distribution of FAPI-therapeutic radioligands [36]. While conceptually related to our work, key differences exist. We used wild-type cell lines for our tumor model, whereas Galbiati et al. used transfected cell lines with higher FAP-receptor density. This required larger ligand doses (up to 18 nmol) to achieve receptor saturation and consequently reduced the impact of the injected mass on uptake in the healthy tissues and blood. In contrast, our study, using a tumor model with lower receptor density, identified optimal ligand doses to maximize tumor uptake while minimizing off-target uptake. Our study is the first to comprehensively evaluate the influence of sFAP levels on the pharmacological performance of FAPI-radiopharmaceuticals, providing novel insights into the balance between therapeutic efficacy and background distribution.

The FAP-positive organs are not the only reason for the high background uptake. High blood pool uptake is caused by sFAP in the plasma of both PC3 and healthy mice. No significant differences were found between the two groups, suggesting that FAP circulation is not correlated to FAP overexpression at tumor stroma. This was similarly observed in the plasma of healthy human volunteers [37], indicating the presence of sFAP in human blood regardless of tumor presence (Fig. 7B). Notably, no human FAP was detected in the plasma. Studies have identified significant levels of sFAP in the blood of healthy individuals. Javidroozi et al. further reported that sFAP levels vary with cancer type and progression. Their findings revealed significantly lower plasma sFAP levels in patients with gynecologic, hematologic, lung, and head-and-neck cancers compared to healthy controls, but no significant differences in prostate cancer, aligning with our observations [38].

The highest blood and FAP-positive organs uptake was observed at the lowest injected masses (10 pmol for biodistribution and 100 pmol for imaging), while increasing the injected doses, their uptake was gradually decreased and eventually reached a law plateau at saturation levels above 600 pmol. This is a key finding, as it allows identification of an optimum mass dose for the FAPI-radiotracers by balancing the increasing tumor uptake, at doses of 350–600 pmol, with the decreasing background uptake in other organs and blood. Increasing the mass from 10 pmol to the respective optimal dose for higher tumor uptake, blood uptake could be reduced by 5–8 fold, raising tumor-to-blood ratios from 1 to 10, with monomers outperforming dimers (Fig. 5, S8). This pattern was consistent for all radiotracers, enhancing PET imaging contrast and reducing radiation exposure to healthy tissue in long-term therapies. One possible explanation for this might be the lower density of FAP receptors in healthy organs and blood compared to the tumor. Consequently, the receptors in these organs are saturated by the unlabeled compound at lower injected doses than those required for the saturation of the tumor.

A closer examination of the in vivo performance of each radiotracer separately, along with a comparative analysis between them, can provide additional valuable insights. All three ^68^Ga-labeled FAPI-dimers at early time-points, i.e. 1 h p.i., exhibit higher background uptake compared to the monomers, even at an injected mass of 600 pmol. However, it shall be noted, that in the case of ^177^Lu-labelled dimers the early high background uptake is reduced with time [4, 19, 28].

[^68^Ga]Ga-DOTAGA.(SA.FAPI)_2_ shows the highest background uptake values across all tested doses. This may be attributed to its stronger lipophilic character compared to [^68^Ga]Ga-DO3A.Glu.(FAPI)_2_ and [^68^Ga]Ga-DOTAGA.Glu.(FAPI)_2_. The gradual increase of the injected mass greatly influences the pharmacokinetics of the radiotracers. [^68^Ga]Ga-DOTAGA.(SA.FAPI)_2_ and [^68^Ga]Ga-DO3A.Glu.(FAPI)_2_, the two more lipophilic dimeric tracers, show a completely different in vivo profile at higher injected masses, exhibiting significantly increased uptake, especially in excretory organs such as the liver and kidneys. Generally, pharmacokinetic profiles are very complicated, and explaining changes in excretion pathways can be difficult and uncertain. It is unlikely that only radioligand hydrophilicity causes pharmacokinetic differences. Those differences result from a complex interaction of factors like hydrophilicity, charge, protein binding affinity, and molecular structure [39, 40]. [^68^Ga]Ga-DATA^5m^.SA.FAPI provides evidence of this phenomenon. Despite being the most hydrophilic tracer, and thus expected to be primarily eliminated through the kidneys into the urine, it demonstrates substantial hepatic metabolism at concentrations up to 600 pmol. This tracer is rapidly excreted via the gallbladder and ultimately into the intestine. However, at higher doses, residual activity in the liver is observed, likely due to saturation of the metabolic process.

In subsequent experiments, the impact of DDP4 and PREP on the selectivity of FAP-radiotracers was extensively investigated in vivo, to determine the extent of any non-specific uptake. Previous in vitro studies have demonstrated that all the radiotracers included in this work exhibit selectivity against the closely related serine proteases of the S9 family (PREP, DPP4, DPP8, and DPP9) [21–24]. This selectivity is crucial for their in vivo applicability, as recognition of these enzymes by FAP-radiotracers could compromise tumor selectivity and reduce overall in vivo performance. After confirming the presence of only intact radiotracers in blood circulation by metabolite analysis in mouse blood, we further investigated their in vivo selectivity against PREP and DPP4 in both healthy and PC3-mice. Only the co-injection of [^68^Ga]Ga-DOTAGA.Glu.(FAPI)_2_, either with DOTAGA.Glu.(FAPI)_2_ alone or with the PREP or DDP4 inhibitors in several combinations, resulted in reduced background uptake. These along with previous in vitro published data clearly indicate that DOTAGA.Glu.(FAPI)_2_ is highly selective for FAP [21]. Furthermore, pancreatic uptake increased when [^68^Ga]Ga-DOTAGA.Glu(FAPI)_2_ was co-injected with the PREP- and DPP4-inhibitors. The underlying mechanism responsible for this observation remains unclear at present. Given that PREP is also reported to be present in tumors [41, 42], [^68^Ga]Ga-DOTAGA.Glu.(FAPI)_2_ was also co-injected with PREP and DPP4 inhibitors in PC3-mice without affecting tumor uptake or overall pharmacokinetics.

The presence of sFAP poses significant challenges, complicating efforts to predict how FAPI-radiopharmaceuticals will behave in the body. This could affect both the distribution and elimination of the radiopharmacuticals, impacting overall diagnostic and therapeutic outcomes. Researchers and clinicians must account for the impact of sFAP when designing and administering these radiopharmaceuticals. The potential interference by sFAP necessitates careful consideration of dosing strategies and may require adjustments to optimize the therapeutic efficacy and diagnostic accuracy of FAPI-radiopharmaceuticals [43].

The proof-of-principle preliminary human studies clearly confirmed the arguments of preclinical observations. Similar studies have to be performed with dimers to further consolidate the above findings, as it would have huge impact in the therapy settings.

In summary, the extensive dose escalation study on monomeric and dimeric FAPI-radiotracers highlighted the critical importance of precise mass administration. The findings revealed that achieving optimal in vivo performance does not depend only on the presence of sFAP in the bloodstream, but also on administering the correct mass of the radiotracer. Achieving the desired diagnostic and therapeutic outcomes requires a careful balance in the administration process, ensuring that FAPI-radiotracers function favorably for maximum efficacy.

## Electronic supplementary material

Below is the link to the electronic supplementary material.

Supplementary Material 1

## Data Availability

All data generated or analyzed during this study are included in this published article and its supplementary information files.
