# Supplementary material for: Improved FAPI-radiopharmaceutical pharmacokinetics from the perspectives of a dose escalation study
Source: Eur J Nucl Med Mol Imaging. 2025 Feb 26;52(9):3238–51. doi: 10.1007/s00259-025-07141-1 (PMC12222399; doi:10.1007/s00259-025-07141-1)
Supplement: Supplementary file 1 — Supplementary Material 1 [file 259_2025_7141_MOESM1_ESM.docx]

**Supplemental Data**

**Improved FAPI-radiopharmaceutical pharmacokinetics from the perspectives of a dose escalation study**

Adrianna Bilinska^1^, Sanjana Ballal^2^, Chandrasekhar Bal^2^, Tilman Läppchen^1^, Eirinaios Pilatis^1^, Elena Menéndez^1^, Euy Sung Moon^3^, Marcel Martin^3^, Frank Rösch^3^, Axel Rominger^1^, Eleni Gourni^1^

^1^Department of Nuclear Medicine, Inselspital, Bern University Hospital, Bern, Switzerland

^2^Department of Nuclear Medicine, All India Institute of Medical Sciences, New Delhi, India

^3^Department of Chemistry–TRIGA site, Johannes Gutenberg-University Mainz, Mainz, Germany

**Reagents and instrumentation**

All reagents were of the best grade available and were purchased from common suppliers. All culture reagents were from Gibco BRL, Life Technologies (Grand Island, NY). The human prostate adenocarcinoma cell line PC3 (CLS-300312, Lot number 816SF) and cancer associated prostate fibroblast cell line (hTERT PF179T, ATCC CRL-3290, Lot Number 0303) were obtained from Cell Lines Service GmbH (CLS, Eppelheim, Germany) and the American Type Culture Collection (ATCC, Manassas, Virginia), respectively. The human serum was commercially available by Sigma (H4522). The GalliaPharm® Ge-68/Ga-68 generator was available from Eckert & Ziegler (Berlin, Germany). The quality control of the precursor and the radiotracers was performed by an analytical Reverse-Phase High Performance Liquid Chromatography (RP-HPLC) on an analytical Nucleosil 120-5 column C18 (250 x 4.5 mm) applying a linear gradient of 15-90% solvent B in 30 min at a flow rate of 1 mL/min. (solvent A, 0.1% trifluoroacetic acid (TFA)/water (H_2_O); solvent B, 0.1%TFA/acetonitrile (ACN). Ultraviolet detection was performed using an Agilent detector at 214 nm. For radioactivity measurement, a Na(TI) well-type scintillation Gina star was used. The radiotracer solutions for the experiments were prepared by dilution with 0.9% NaCl (Bichsel AG, Interlaken, Switzerland).

Quantitative γ-counting was performed with a COBRA II γ-system well counter from Packard Instrument (USA). For μPET/CT studies, a dedicated micro-PET/SPECT/CT scanner (Albira Si; Bruker Biospin, Ettlingen, Germany) was used.

Mice were purchased from Janvier Labs (Rue du Genest, 53940 Le Genest-Saint-Isle, France), pentobarbital natrium (150mg/kg) from Streuli Pharma SA (Uznach, Switzerland).

**Radiochemistry / Quality control of the radiotracers**

[^68^Ga]Ga-DOTA.SA.FAPI, [^68^Ga]Ga-DATA^5m^.SA.FAPI, [^68^Ga]Ga-DO3A.Glu.(FAPI)_2_, [^68^Ga]Ga-DOTAGA.(SA.FAPI)_2_ and [^68^Ga]Ga-DOTAGA.Glu.(FAPI)_2_ were prepared within 5 min, using the Modular-Lab PharmTracer module by Eckert & Ziegler (Berlin, Germany). The radiolabeling performance of all precursors was assessed at pH 4.6 with 1 M HEPES buffer at 90 °C for 5 min using, in each case, the minimum conjugate amount. Briefly, the Ge-68/Ga-68 generator was eluted with 5 mL 0.1 N HCl and the eluate (~300 MBq) was loaded onto a cation exchange column (Strata-XC, Phenomenex). Gallium-68 was eluted with 700 μL of a mixture of 5.5 M NaCl / 0.1 M HCl (prepared by mixing 0.530 mL of ultrapure 30% aq. HCl and 49.5 mL of 5.5M NaCl) directly into a vial containing 2 mL 1 M HEPES (pH 5.3), 200 μL of EtOH, and 20 μg (approximately 20 nmol) of the conjugate, followed by SepPak C-18 purification to remove uncomplexed gallium-68. The purified [^68^Ga]Ga**-**DOTAGA.Glu.(FAPI)_2_, [^68^Ga]Ga**-**DO3A.Glu.(FAPI)_2_, [^68^Ga]Ga**-**DOTAGA.(SA.FAPI)_2_, [^68^Ga]Ga**-**DOTA.SA.FAPI and [^68^Ga]Ga**-**DATA^5m^.SA.FAPI were eluted in 1.4 mL of a solution of 6.2% EtOH in PBS. The % of EtOH in the formulations for the in vivo studies was varying between 0.1 and 5%.

The quality control of [^68^Ga]Ga-DOTA.SA.FAPI, [^68^Ga]Ga-DATA^5m^.SA.FAPI, [^68^Ga]Ga-DO3A.Glu.(FAPI)_2_, [^68^Ga]Ga-DOTAGA.(SA.FAPI)_2_ and [^68^Ga]Ga-DOTAGA.Glu.(FAPI)_2_ was performed by radio-HPLC as described in the reagents and instrumentation. The presence of free gallium-68 and ^68^Ga-labelled colloid in the [^68^Ga]Ga-DOTA.SA.FAPI, [^68^Ga]Ga-DATA^5m^.SA.FAPI, [^68^Ga]Ga-DO3A.Glu.(FAPI)_2_, [^68^Ga]Ga-DOTAGA.(SA.FAPI)_2_ and [^68^Ga]Ga-DOTAGA.Glu.(FAPI)_2_ preparations was quantified by radio thin layer chromatography (Radio-TLC) using ITLC-SG-plates (Glass microfiber chromatography paper impregnated with silica gel) and two different mobile phase eluents: a) 0.1 M Na-citrate; b) MeOH / 1 M ammonium acetate (1/1, v/v).

**Lipophilicity**

The lipophilicity (LogD_octanol/PBS,_ pH 7.4) of [^68^Ga]Ga-DOTA.SA.FAPI, [^68^Ga]Ga-DATA^5m^.SA.FAPI, [^68^Ga]Ga-DO3A.Glu.(FAPI)_2_, [^68^Ga]Ga-DOTAGA.(SA.FAPI)_2_ and [^68^Ga]Ga-DOTAGA.Glu.(FAPI)_2_ was estimated by the “shake-flask” method: The labelled conjugate (20 pmol; 0.3 MBq) was added to a 1:1 mixture of 1-octanol (500 μL) and PBS (500 μL, pH 7.4). The mixture was intermittently vortexed for 1 h to reach the equilibrium and then centrifuged (3000 rpm) for 10 min. From each phase, an aliquot (100 μL) was pipetted out and measured in a gamma-counter. Each measurement was repeated five times. Care was taken to avoid cross-contamination between the phases. The partition coefficient was calculated as the average log ratio of the radioactivity in the organic fraction and the PBS fraction.

**Cell lines / Animal Models**

The human prostate adenocarcinoma cell line PC3 (RRID:CVCL_0035) and the cancer associated prostate fibroblast cell line CAF (hTERT PF179T CAF) isolated from the prostate of a male patient with cancer were used in the present study. The human prostate adenocarcinoma cell line PC3 (RRID:CVCL_0035) was cultured in Dulbecco's Modified Eagle Medium (DMEM) with low glucose (1g/l): F-12 Nutrient Mix with GlutaMAX™-I (1:1 mixture ratio). The cancer associated prostate fibroblast cell line CAF was cultured in the same conditions with Eagle's Minimal Essential Medium (EMEM) supplemented with sodium bicarbonate 1500 mg/L and puromycin 1 μg/mL. In each case, the medium was supplemented with 10% fetal bovine serum (FBS), penicillin (100 U/mL) and streptomycin (100 µg/mL). All the cell lines were cultured at 37°C and 5% CO_2_.

PC3 prostate cancer cells were used to develop tumor models in male athymic Balb/C nude mice (CByJ.Cg-Foxn1nu/J) (6 weeks, 20-25 g) (RRID:IMSR_JAX:002019**)**. Mice were implanted with PC3 cells (3.5x10^6^/100 µL PBS) into the right shoulder, and used for biodistribution and PET/CT once tumors reached 250-300 mm³. For imaging and biodistribution experiments, mice were randomly assigned to groups based on their tumor size to ensure that each group has a similar size distribution. Healthy male athymic Balb/C mice were used for *in vivo* selectivity and RNA extraction (Animal License: BE98/2021).

**Metabolic Stability**

Approximately 1 mL of blood was collected to heparinized-tubes from PC3-xenografts 10 minutes after intravenous injection of 600 pmol of [^68^Ga]Ga-DOTA.SA.FAPI, [^68^Ga]Ga-DATA^5m^.SA.FAPI, [^68^Ga]Ga-DO3A.Glu.(FAPI)_2_, [^68^Ga]Ga-DOTAGA.(SA.FAPI)_2_ and [^68^Ga]Ga-DOTAGA.Glu.(FAPI)_2_ (total volume: 100 µL of NaCl 0.9 %). Blood collection was followed by plasma separation by centrifugation at maximum speed for 10 min at 4 ºC. Subsequently, plasma was treated with 1:1 solution of MeOH:Acetonitrile in order to precipitate the proteins. After centrifugation at maximum speed for 10 min at 4 ºC, supernatant was injected to RP-HPLC.

**Biodistribution Study - Dose Escalation**

PC3 tumor bearing mice were used between the 15th and 17th day after the implantation and when the tumor size was 250 ± 90 mm^3^. Mice were injected intravenously with 10-1500 pmol (0.03-4 MBq/100 µL) of all radiotracers. One hour p.i., the mice were euthanized for biodistribution studies (n=3). For the 1000 and 1500 pmol doses, formulations included 600 pmol of each labeled radiotracer with 400 or 900 pmol of the respective non-labeled precursor. All animals were terminally anesthetized by intraperitoneal injection of an overdose of pentobarbital natrium (150mg/kg; Streuli Pharma SA) at 1 h after injection of the radiotracers. The organs of interest were dissected and weighted, and the radioactivity in tissue samples was counted in a γ-counter. Biodistribution data are given as percent of injected activity per gram of tissue (% IA/g) and are means ± SD (n = 3).

**Small-Animal PET/CT Imaging**

PET/CT images were obtained upon injection of: 100, 350, 600, 1000, and 1500 pmol of each of the ^68^Ga-labeled radiotracers (0.3-4 MBq/100 μL) in PC-mice at 1 h p.i..

PET/CT scans were acquired using a dedicated micro-PET/SPECT/CT scanner (Albira Si; Bruker Biospin, Ettlingen, Germany). Static PET images were obtained 1 hour after injection of 100 - 1500 pmol (0.3 - 4 MBq / 100 uL) of [^68^Ga]Ga**-**DOTAGA.Glu.(FAPI)_2_, [^68^Ga]Ga**-**DO3A.Glu.(FAPI)_2_, [^68^Ga]Ga**-**DOTAGA.(SA.FAPI)_2_, [^68^Ga]Ga**-**DOTA.SA.FAPI and [^68^Ga]Ga**-**DATA^5m^.SA.FAPI on a PC3 xenograft mouse model (n=3/group). The mice were scanned for 20 – 30 minutes, under 2% isoflurane anesthesia as a mixture of O_2_ with a 0.8 LPM flow. During the scan, respiration rate was 70 – 100 breaths / min, with an external temperature of 35 – 36 ^o^C.

Reconstruction of PET data employed 1 iteration of the Ordered Subset Expectation Maximization (OSEM) algorithm with a voxel size of 0.75 mm for image quantification and 12 iterations of Maximum a Posteriori (MAP) with a voxel size of 0.25mm for the Maximum Intensity Projection (MIP) extraction. In addition, Partial Volume Effect (PVE) correction and Point Spread function (PSF) deconvolution were applied. CT step and shoot scan was carried out using an X-ray tube voltage of 45 kV and an intensity of 400 uA. A Filtered Back - Projection (FBP) algorithm was utilized as a reconstruction method with a voxel size of 0.25 mm.

PMOD 4.404 imaging suite (PMOD; PMOD Technologies LLC) allowed quantification of the radioligand accumulation within the organs and the presentation of the MIP. The reconstructed data were decay corrected upon the start of the PET scan for the Gallium-68 decay. The color scale of the MIP was set from 0 to 12 % IA/g and a Gaussian 3D algorithm with a 1 mm isotropic kernel applied, providing a qualitative comparison among the images.

**RNA Extraction**

Organs of interest from of healthy mice (n=2) and PC3 xenografts (n=2) were collected to 500 µL of RNAlater® solution (ThermoFisher Scientific). Approximately 1 mL of blood was collected to heparinized tubes. DNA/RNA Extracol Kit and Universal Blood RNA Purification Kit (EurX) were used in order to extract total RNA from selected tissues. The procedure of extraction followed manufacturer’s protocol. The concentration and purity of the extracted RNA were assessed using a NanoDrop 2000 spectrophotometer (ThermoFisher Scientific), with 1 µL of extracted RNA.

**Real Time PCR**

One microgram of RNA was reversely transcribed using a High-Capacity cDNA Reverse Transcription Kit (Applied Biosystems) following manufacturers protocol. Primer sets for quantitative real-time PCR for FAP and reference genes were designed using the PRIMER 3 software (<http://simgene.com/Primer3>),based on mouse genome sequence: GRCm39 (*FAP* – GenBank ID:14089).

All primer sets were tested in a gradient PCR to assess their specificity and annealing temperature (Table S9). The relative transcript levels were assessed using a Hot FIREPol EvaGreen qPCR Mix (Solis Biodyne) with QuantStudio 7 Real-Time PCR System (ThermoFisher Scientific). Standard curves were designed as tenfold dilutions of the PCR products. Relative transcript levels of all samples were calculated after normalization with the transcript level of reference genes of GAPDH and RLPL0. All samples were analyzed in triplicate.

**ELISA**

Approximately 1 mL of blood was collected from PC3 tumor bearing mice (n=6), healthy mice (n=4) and healthy human volunteers (n=4) to heparin-coated tubes (Heparin 100 IE/ml), followed by plasma separation. Cell culture media from CAFs was collected after 10 days of culture in T75 flask without media change. ELISA was performed using the Human and Mouse DuoSet ELISA Development System (R&D Systems) using plasma and cell culture media samples following the manufacturer protocol. The samples absorbance was measured using Tecan Sunrise (Tecan) plate reader at 450 nm. All experiments were carried out two times in triplicate.

***In vivo* Selectivity of the Radiolabeled Tracers towards FAP**

Competition experiments were performed in 8 cohorts of healthy mice. Ten pmol of [^68^Ga]Ga-DOTAGA.Glu.(FAPI)_2_ (0.08-0.09 MBq/100 μL) was co-injected intravenously with varying combinations of 500 pmol of DOTAGA.Glu.(FAPI)_2_, 3 different PREP (Salidroside Baicalin, KYP-2047) and a DPP4 (DPP IV) inhibitor. The PREP and DPP4 inhibitors were commercially available: DPP4 inhibitor (Sigma Aldrich, Millipore, Germany), Baicalin (Sigma Aldrich, Germany), Salidroside (Lubio Science, Switzerland), KYP-2047 (Sigma Aldrich, Germany). All inhbitors were diluted to final concentration 500 pmol following manufacturer leads. Biodistribution studies were conducted 1 h p.i..

In a separate group of PC3 tumor bearing mice, the injected mass of [^68^Ga]Ga-DOTAGA.Glu.(FAPI)_2_ which led to the highest tumor uptake, 600 pmol (~4 MBq/100 μL), was co-injected intravenously with 500 pmol of each of the DPP4 and PREP (Salidroside) inhibitors. Biodistribution studies were conducted 1 h p.i.. All animals were terminally anesthetized by intraperitoneal injection of an overdose of pentobarbital natrium (150mg/kg; Streuli Pharma SA) at 1 h after injection of the radiotracers. The organs of interest were dissected and weighted, and the radioactivity in tissue samples was counted in a γ-counter. Biodistribution data are given as percent of injected activity per gram of tissue (% IA/g) and are means ± SD (n = 3).

**More specifically:**

The in vivo selectivity studies were conducted using 10 pmol of the radioligand, along with various combinations of FAP, PREP, and DPP4 inhibitors. Below, an overview of the cohorts utilized in the experimental setup is provided:

**Healthy mice cohorts:**

10 pmol FAPI-radiotracer (baseline control)

10 pmol FAPI-radiotracer + 500 pmol DPP4 inhibitor

10 pmol FAPI-radiotracer + 500 pmol PREP inhibitor (Salidroside)

10 pmol FAPI-radiotracer + 500 pmol PREP inhibitor (KYP-2047)

10 pmol FAPI-radiotracer + 500 pmol PREP inhibitor (Baicalin)

10 pmol FAPI-radiotracer + 500 pmol Salidroside + 500 pmol DPP4 inhibitor

10 pmol FAPI-radiotracer + FAP inhibitor

10 pmol FAPI-radiotracer + FAP inhibitor + 500 pmol Salidroside + 500 pmol DPP4 inhibitor

10 pmol of the FAPI-radiotracer were selected for these experiments because this dose consistently resulted in the highest background uptake. For the competition studies, we used 500 pmol of each inhibitor, as this concentration provided optimal pharmacokinetic performance under our experimental conditions.

In addition, we investigated whether the co-presence of PREP, DPP4, and FAP in our experimental tumor model influenced tumor uptake of the FAPI-radiotracer. To address this, we conducted an additional set of experiments with the following conditions:

**PC3 tumor bearing mice cohorts:**

One cohort received 600 pmol of the FAPI-radiotracer, which led to the highest tumor uptake.

Another cohort received 600 pmol of the FAPI-radiotracer in combination with 500 pmol Salidroside and 500 pmol DPP4 inhibitor.

**Statistical Analysis**

To evaluate the statistical significance of biodistribution data for each radiopharmaceutical across various organs, a two-way ANOVA test was performed. The biodistribution results for different concentrations, derived from a dose escalation, were compared to a reference concentration of 10 pmol.

A two-way ANOVA was conducted to evaluate *in vivo* selectivity by analyzing the variations in inhibitor combinations, using 10 pmol of [^68^Ga]Ga-DOTAGA.Glu.(FAPi)_2_ as the reference. This method was also employed to compare the relative FAP transcript levels in different organs between PC3 xenografts and healthy mice.

All statistical analyses were performed in Prism 8 software (GraphPad Software) to determine statistical significance at the 95% confidence level, with a P-value of less than 0.05 was considered significantly different.


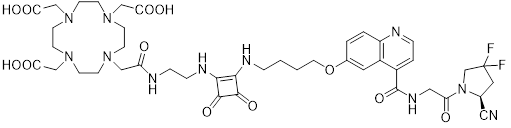


**DOTA.SA.FAPI**

**DATA^5m^.SA.FAPI**

**DO3A.Glu.(FAPI)_2_**

**DOTAGA.Glu.(FAPI)_2_**

**DOTAGA.(SA.FAPI)_2_**

**Figure S1**. Schematic representations of DOTA.SA.FAPI (monomer), DATA^5m^.SA.FAPI (monomer), DO3A.Glu.(FAPI)_2_ (dimer), DOTAGA.Glu.(FAPI)_2_ (dimer) and DOTAGA.(SA.FAPI)_2_ (dimer).

**Results**

**Quality control of the radiotracer/Stability**

Based on the amount of the precursor which was used for the radiolabeling and assuming we lose about 20% during the labeling, the apparent molar activities (A_m_) were in the range of 9 to 22 GBq/µmol (not decay corrected).

With regard to the detection of the formation of colloids by ITLC, using the first radio-TLC eluent, [^68^Ga]Ga**-**DOTAGA.Glu.(FAPI)_2_, [^68^Ga]Ga**-**DO3A.Glu.(FAPI)_2_, [^68^Ga]Ga**-**DOTAGA.(SA.FAPI)_2_, [^68^Ga]Ga**-**DOTA.SA.FAPI and [^68^Ga]Ga**-**DATA^5m^.SA.FAPI and ^68^Ga-labelled colloid remain immobilized at the starting point, whereas free gallium-68 moves with the mobile phase. When the second eluent is used, only the [^68^Ga]Ga-DOTA.SA.FAPI and [^68^Ga]Ga-DOTAGA.(SA.FAPI)_2_ move with the mobile phase / solvent front.


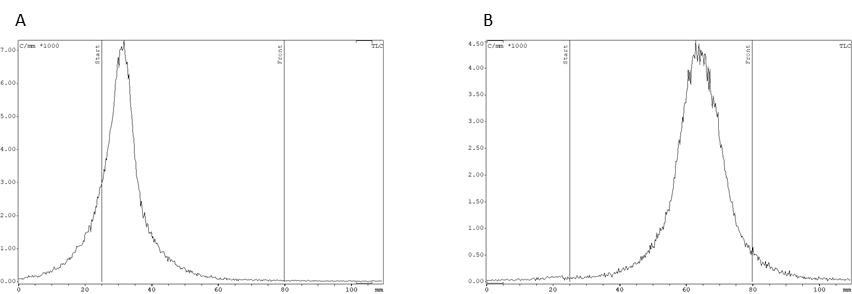


**Figure S2.** Representative TLC profiles in (A) Sodium Citrate and (B) Ammonium Acetate:MeOH of [^68^Ga]Ga-DOTA.SA.FAPI.


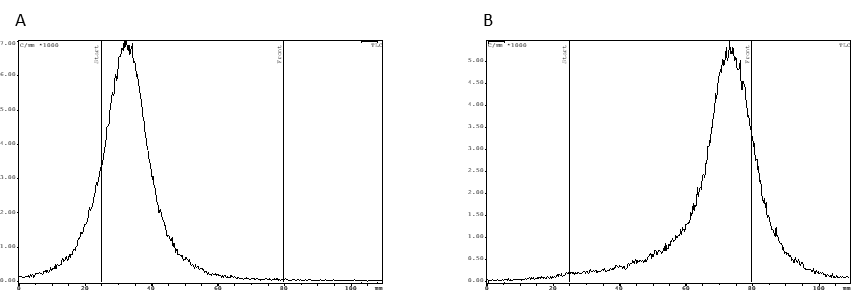


**Figure S3**. Representative TLC profiles in in (A) Sodium Citrate and (B) Ammonium Acetate:MeOH of [^68^Ga]Ga-DATA^5m^.SA.FAPI.


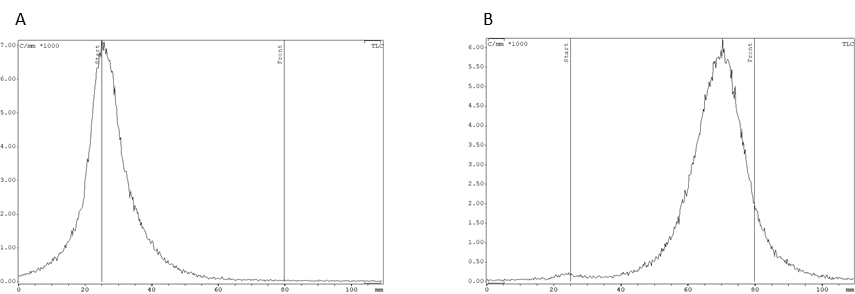


**Figure S4**. Representative TLC profiles in in (A) Sodium Citrate and (B) Ammonium Acetate:MeOH of [^68^Ga]Ga-DO3A.Glu.(FAPI)_2._

.
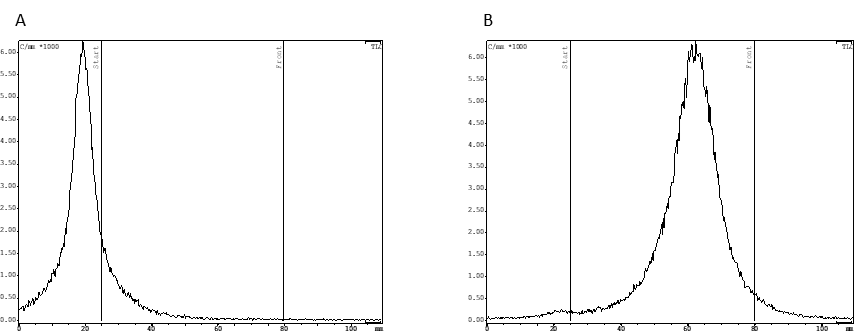


**Figure S5.** Representative TLC profiles in (A) Sodium Citrate and (B) Ammonium Acetate:MeOH of [^68^Ga]Ga-DOTAGA.Glu.(FAPI)_2._

**
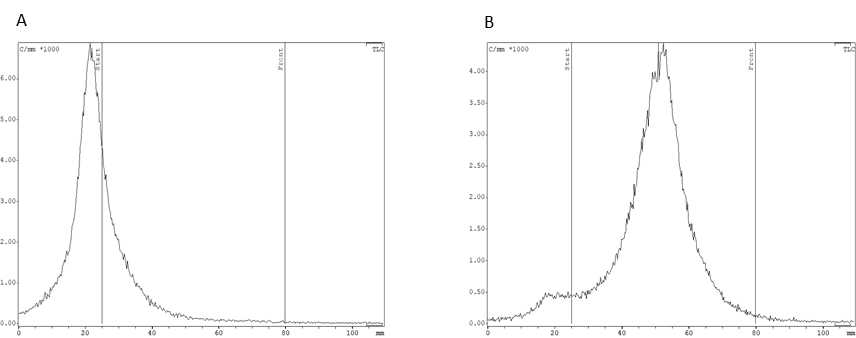
**

**Figure S6**. Representative TLC profiles in in (A) Sodium Citrate and (B) Ammonium Acetate:MeOH of [^68^Ga]Ga-DOTA.(SA.FAPI)_2._


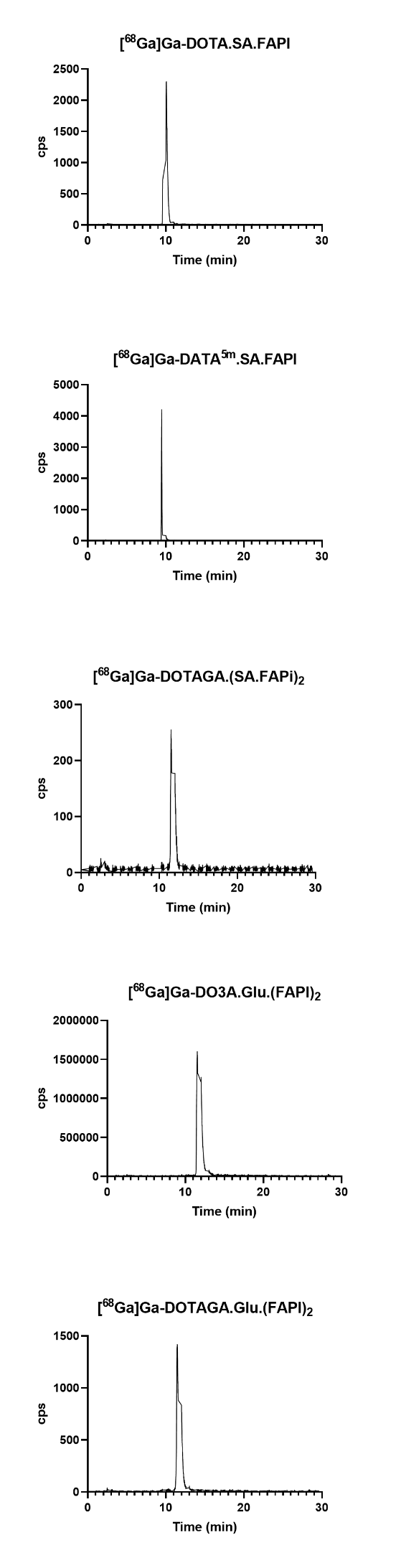


**Figure S7**. Representative radio-HPLC chromatograms of [^68^Ga]Ga-DOTA.SA.FAPI, [^68^Ga]Ga-DATA^5m^.SA.FAPI, [^68^Ga]Ga-DO3A.Glu.(FAPI)_2_, [^68^Ga]Ga-DOTAGA.Glu.(FAPI)_2_, [^68^Ga]Ga-DOTAGA.(SA.FAPI)_2_

**Table S1.** Biodistribution data of [^68^Ga]Ga-DOTA.SA.FAPI.

For the statistical analysis, the comparisons were made between all the injected mass of radiopharmaceutical and the mass of 10 pmol that serves as a reference. The table should be read row-wise. The same letters indicate the absence of significant difference (at the 0.05 level) between the injected masses. Data are presented as % IA/g of tissue and are presented as mean ± SD (*n*=3). SD – standard deviation.

|  | **Organs** | **Injected mass (pmol)** | | | | | |
| --- | --- | --- | --- | --- | --- | --- | --- |
| **[^68^Ga]Ga-DOTA.SA.FAPI** |  | **10** | **100** | **350** | **600** | **1000** | **1500** |
|  | blood  heart  gallbladder  liver  spleen  lung  kidney  stomach  intestine  adrenal  pancreas  muscle  bone  tumor | 8.5±0.6^a^  4.6±1.6^a^  1.8±0.4^a^  2.4±0.1^a^  2.5±0.1^a^  3.5±0.6^a^  3.6±0.3^a^  2±0.4^a^  2.7±0.2^a^  5.5±4.1^a^  8.9±1^a^  2.5±0.2^a^  3.4±0.4^a^  6.1±0.6^a^ | 3.6±0.1^b^  1.4±0.2^b^  4.8±1.8^b^  2.5±0.2^a^  2.5±0.5^a^  1.8±0.1^a^  2.2±0.1^a^  1.1±0.6^a^  1.4±0.2^a^  5.2±0.7^b^  2.8±0.1^b^  2.7±0.3^a^  3.6±1.3^a^  8.8±1.1^b^ | 1.7±0.1^b^  0.6±0.1^b^  4.3±0.8^b^  2.9±0.5^a^  1.5±0.3^a^  1.2±0.1^b^  2.9±0.7^a^  0.6±0.01^a^  0.9±0.1^b^  2.9±1^b^  1.3±0.1^b^  1.2±0.34^a^  5.7±0.8^b^  15.8±3.1^b^ | 1±0.1^b^  0.4±0.1^b^  4.1±0.7^b^  1.2±0.3^a^  0.3±0.1^b^  1±0.8^b^  2.7±0.8^a^  0.4±0.1^a^  0.8±0.2^b^  1±0.1^b^  0.6±0.04^b^  0.7±0.2^b^  3.3±0.4^a^  8.8±1.8^b^ | 0.7±0.1^b^  0.3±0.04^b^  2.9±1.5^a^  1.4±0.3^a^  0.4±0.02^b^  1±0.2^b^  3.9±1.6^a^  0.3±0.2^b^  0.6±0.1^b^  0.4±0.04^b^  0.4±0.1^b^  0.3±0.1^b^  1.6±0.6^b^  4.6±0.2^a^ | 0.8±0.1^b^  0.4±0.1^b^  7±3.5^b^  1.5±0.1^a^  0.4±0.04^b^  1.1±0.1^b^  3.9±0.4^a^  0.5±0.2^a^  1±0.02^b^  1.4±0.6^b^  0.4±0.03^b^  0.4±0.1^b^  1.4±0.4^b^  4.1±2.1^b^ |

**Table S2.** Biodistribution data of [^68^Ga]Ga-DATA^5m^.SA.FAPI.

For the statistical analysis, the comparisons were made between all the injected mass of radiopharmaceutical and the mass of 10 pmol that serves as a reference. The table should be read row-wise. The same letters indicate the absence of significant difference (at the 0.05 level) between the injected masses. Data are presented as % IA/g of tissue and are presented as mean ± SD (*n*=3). SD – standard deviation.

|  | **Organs** | **Injected mass (pmol)** | | | | | |
| --- | --- | --- | --- | --- | --- | --- | --- |
| **[^68^Ga]Ga-DATA^5m^.SA.FAPI** |  | **10** | **100** | **350** | **600** | **1000** | **1500** |
|  | blood  heart  gallbladder  liver  spleen  lung  kidney  stomach  intestine  adrenal  pancreas  muscle  bone  tumor | 8.2±0.7^a^  3.2±0.4^a^  17±5.8^a^  2.7±0.4^a^  2.1±0.3^a^  3.6±0.7^a^  3.2±0.5^a^  2±0.4^a^  4.2±0.8^a^  8.3±0.8^a^  7.4±1.9^a^  2±0.3^a^  3.2±0.9^a^  5±0.6^a^ | 4.8±0.7^a^  2±0.3^a^  38±11.6^b^  2.6±0.1^a^  1.4±0.2^a^  2.7±0.4^a^  2.8±0.4^a^  1.7±0.3^a^  4.3±2.1^a^  3.1±1.1^a^  5.6±1.2^a^  3.6±0.3^a^  3.3±0.7^a^  11.1±0.4^b^ | 1.9±0.1^b^  0.8±0.02^a^  44.4±8.7^b^  2.8±0.1^a^  0.5±0.1^a^  1.3±0.2^a^  1.7±0.02^a^  0.7±0.1^a^  2.9±1.1^a^  3.4±0.9^a^  1.7±0.1^b^  1.6±0.2^a^  2.9±0.5^a^  12±2.1^b^ | 1.2±0.1^b^  0.5±0.1^a^  22.4±3.1^b^  3±0.6^a^  0.4±0.04^a^  1±0.2^a^  1.8±0.5^a^  0.6±0.1^a^  2.5±1^a^  2.6±0.1^a^  1.1±0.1^b^  0.9±0.3^a^  2.5±0.7^a^  10.4±3.3^b^ | 1±0.2^b^  0.4±0.1^a^  31.2±6.5^b^  4.6±0.9^a^  0.4±0.1^a^  0.9±0.2^a^  2.3±0.6^a^  0.6±0.2^a^  3.9±1.5^a^  1.6±0.3^a^  0.8±0.1^b^  0.6±0.02^a^  1.9±0.4^a^  8.8±1.4^a^ | 0.8±0.1^b^  0.4±0.06^a^  49±6.3^b^  4.5±0.6^a^  0.3±0.04^a^  1±0.3^a^  2±0.5^a^  0.5±0.2^a^  2.2±0.6^a^  1.1±0.2^a^  0.7±0.08^b^  0.4±0.04^a^  1.7±0.2^a^  6.3±1.1^a^ |

**Table S3.** Biodistribution data of [^68^Ga]Ga-DOTAGA.(SA.FAPI)_2._

For the statistical analysis, the comparisons were made between all the injected mass of radiopharmaceutical and the mass of 10 pmol that serves as a reference. The table should be read row-wise. The same letters indicate the absence of significant difference (at the 0.05 level) between the injected masses. Data are presented as % IA/g of tissue and are presented as mean ± SD (*n*=3). SD – standard deviation.

|  | **Organs** | **Injected mass (pmol)** | | | | | |
| --- | --- | --- | --- | --- | --- | --- | --- |
| **[^68^Ga]Ga-DOTAGA.(SA.FAPI)_2_** |  | **10** | **100** | **350** | **600** | **1000** | **1500** |
|  | blood  heart  gallbladder  liver  spleen  lung  kidney  stomach  intestine  adrenal  pancreas  muscle  bone  tumor | 23.3±3.8^a^  7.1±0.7^a^  1.7±0.6^a^  8.2±2.1^a^  4.7±0.5^a^  7.1±0.2^a^  8.1±0.6^a^  3.3±0.4^a^  5±0.2^a^  6.5±2.6^a^  11.7±1.7^a^  2.4±0.5^a^  4±0.3^a^  6.3±1^a^ | 11.8±0.3^b^  4.3±0.2^b^  2±0.8^a^  3.1±0.1^b^  2.6±0.2^a^  4.2±0.1^b^  4.6±0.4^b^  3±0.3^a^  3.9±0.9^a^  6.1±2.5^a^  8.7±0.6^b^  2.6±0.1^a^  2.9±0.5^a^  8±0.4^a^ | 7.6±0.3^b^  2.5±0.1^b^  3.3±0.8^a^  3.4±0.4^b^  2.2±0.3^a^  4.8±0.7^a^  6.3±0.7^a^  2.2±0.3^a^  2.7±0.1^a^  8.3±3.3^a^  3.7±0.07^b^  2.2±0.4^a^  3.9±0.4^a^  12.7±2.1^b^ | 5.4±0.6^b^  2.3±0.2^b^  6.1±1.7^b^  6.1±0.8^a^  2.5±0.3^a^  7.5±0.6^a^  10.5±1.1^a^  1.8±0.4^a^  2.2±0.1^b^  12.6±3.1^b^  3.1±0.2^b^  2±0.1^a^  4.4±0.3^a^  14.4±2.7^b^ | 4.9±0.4^b^  1.8±0.3^b^  6.2±1^b^  7.9±0.4^a^  3±0.06^a^  9.4±0.6^a^  13.4±0.6^b^  1.1±1.1^a^  2±0.14^b^  4.9±2^a^  2.9±0.1^b^  1.7±0.1^a^  4.2±0.4^a^  13.3±1.8^b^ | 4.6±0.2^b^  2±0.04^b^  6.4±3^b^  10.7±0.5^a^  3.6±0.3^a^  12.2±0.7^b^  18.5±0.7^b^  1.7±0.2^a^  2.8±0.2^a^  1.5±2.2^b^  2.5±0.3^b^  1.6±0.05^a^  2.9±1.2^a^  12.2±0.7^b^ |

**Table S4.** Biodistribution data of [^68^Ga]Ga-DO3A.Glu.(FAPI)_2._

For the statistical analysis, the comparisons were made between all the injected mass of radiopharmaceutical and the mass of 10 pmol that serves as a reference. The table should be read row-wise. The same letters indicate the absence of significant difference (at the 0.05 level) between the injected masses. Data are presented as % IA/g of tissue and are presented as mean ± SD (*n*=3). SD – standard deviation.

|  | **Organs** | **Injected mass (pmol)** | | | | | |
| --- | --- | --- | --- | --- | --- | --- | --- |
| **[^68^Ga]Ga-DO3A.Glu.(FAPI)_2_** |  | **10** | **100** | **350** | **600** | **1000** | **1500** |
|  | blood  heart  gallbladder  liver  spleen  lung  kidney  stomach  intestine  adrenal  pancreas  muscle  bone  tumor | 16.7±2.4^a^  6.8±0.5^a^  nd  3.6±0.5^a^  3.8±0.3^a^  5±0.7^a^  5.4±0.6^a^  3.3±0.7^a^  4.4±0.6^a^  nd  15.2±2.5^a^  3.2±0.8^a^  10.5±3.6^a^  8.4±0.7^a^ | 7.4±1.5^b^  3.3±0.2^b^  1 ±0.2  2.1±0.3^a^  2.1±0.4^a^  3.7±0.2^a^  3.2±0.4^b^  2.2±0.5^a^  2.7±0.5^a^  nd  8.7±1.2^b^  2.9±0.4^a^  2.2±0.2^b^  9.8±2.2^a^ | 2.3±0.1^b^  1±0.1^b^  0.7±0.4  1.6±0.6^a^  0.9±0.2^b^  2.1±0.04^b^  2.7±0.8^b^  1±0.2^b^  0.9±0.1^b^  3.3±1.2  2.6±0.1^b^  2.2±0.2^a^  3.7±0.9^b^  14.1±0.5^b^ | 2±0.1^b^  1±0.1^b^  2.3±1.8  4.2±1.2^a^  1.3±0.3^b^  3.8±1^a^  6.7±1.7^a^  0.9±0.07^b^  0.9±0.2^b^  3.6±1.1  1.8±0.1^b^  2±0.03^a^  5±3^b^  19±2.4^b^ | 1.7±0.2^b^  0.9±0.3^b^  2.4±1.2  4.3±0.7^a^  1.2±0.2^b^  3.8±0.5^a^  7.1±0.6^a^  0.8±0.02^b^  0.8±0.2^b^  3.6±1.2  1.6±0.1^b^  1.2±0.4^a^  3.1±0.7^b^  14.9±2.7^b^ | 1.6±0.03^b^  0.9±0.1^b^  4.3±2.2  7±0.8^b^  1.9±0.1^a^  5.3±1.2^a^  10.5±0.3^b^  0.9±0.1^b^  1±0.1^b^  5.4±1.3  1.3±0.06^b^  1.1±0.2^b^  2.8±0.2^b^  10.1±1.6^a^ |

**Table S5.** Biodistribution data of [^68^Ga]Ga-DOTAGA.Glu.(FAPI)_2._

For the statistical analysis, the comparisons were made between all the injected mass of radiopharmaceutical and the mass of 10 pmol that serves as a reference. The table should be read row-wise. The same letters indicate the absence of significant difference (at the 0.05 level) between the injected masses. Data are presented as % IA/g of tissue and are presented as mean ± SD (*n*=3). SD – standard deviation.

|  | **Organs** | **Injected mass (pmol)** | | | | | |
| --- | --- | --- | --- | --- | --- | --- | --- |
| **[^68^Ga]Ga-DOTAGA.Glu.(FAPI)_2_** |  | **10** | **100** | **350** | **600** | **1000** | **1500** |
|  | blood  heart  gallbladder  liver  spleen  lung  kidney  stomach  intestine  adrenal  pancreas  muscle  bone  tumor | 25.9±3.2^a^  8.7±1^a^  nd  6.5±1.3^a^  6.1±2^a^  8.5±0.1^a^  10.1±1.9^a^  4.1±0.9^a^  5.7±2.1^a^  11.3±0.7^a^  20.2±2.8^a^  3.8±0.88^a^  4.9±2^a^  12.5±1.7^a^ | 9.4±0.4^b^  4.2±0.07^b^  2.1±1  2.5±0.2^b^  2.3±0.3^b^  3.7±0.3^b^  4.1±0.8^b^  2.5±0.6^a^  2.7±0.5^b^  8.9±2.4^b^  8.3±0.3^b^  2±0.3^a^  2.4±0.9^b^  6.7±2.3^b^ | 3.9±0.2^b^  1.3±0.2^b^  0.7±0.5  1.1±0.09^b^  1±0.07^b^  1.8±0.2^b^  2.9±0.2^b^  1.2±0.07^b^  1.2±0.3^b^  3.3±2^b^  2.6±0.5^b^  1.9±0.2^a^  3.6±0.8^a^  10±0.5^b^ | 3.2±0.5^b^  1.4±0.1^b^  2.3±2.5  2.1±0.2^b^  1.3±0.3^b^  2.7±0.3^b^  6.5±1.1^b^  1.5±0.08^b^  1.4±0.2^b^  3.7±1.3^b^  2.7±0.8^b^  1.6±0.2^a^  4.9±1.3^a^  16.5±2.3^b^ | 2.2±0.3^b^  0.8±0.1^b^  1.9±1  1.7±0.1^b^  0.8±0.1^b^  2.2±0.2^b^  5.3±0.7^b^  0.9±0.2^b^  1±0.1^b^  4.1±0.4^b^  1.4±0.1^b^  0.9±0.06^a^  2.5±0.3^b^  9.7±1.5^b^ | 2.5±0.2^b^  1.1±0.2^b^  4.2±0.8  2.7±0.3^b^  1.2±0.08^b^  3.4±0.2^b^  8.1±0.7^a^  0.9±0.04^b^  1.1±0.2^b^  2.5±0.1^b^  1.3±0.04^b^  0.8±0.04^a^  2.8±0.2^b^  7.8±0.6^b^ |


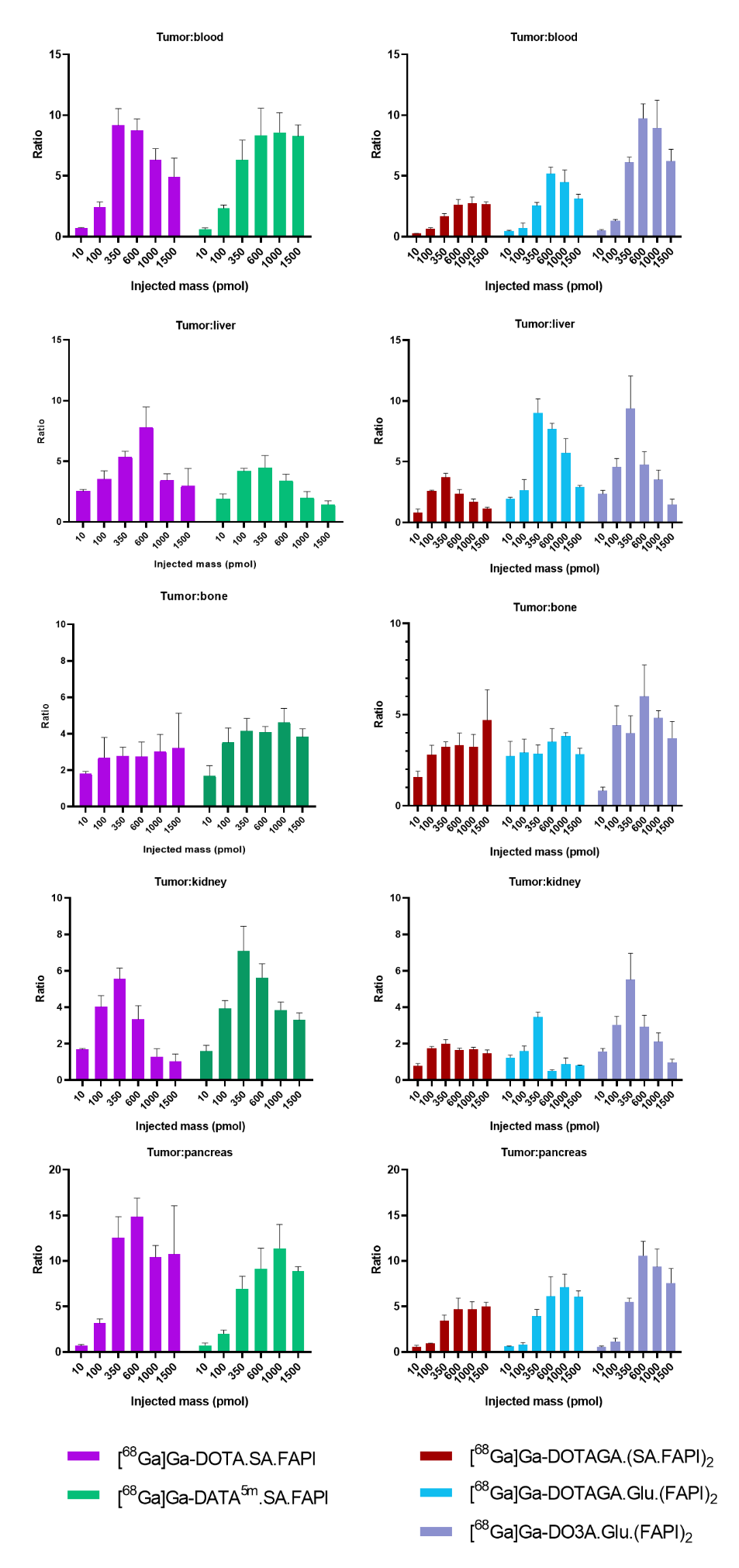


**Figure S8.** Tissue radioactivity ratios of compounds [^68^Ga]Ga-DOTA.SA.FAPI, [^68^Ga]Ga-DATA^5m^.SA.FAPI, [^68^Ga]Ga-DO3A.Glu.(FAPI)_2_, [^68^Ga]Ga-DOTAGA.Glu.(FAPI)_2_, [^68^Ga]Ga-DOTAGA.(SA.FAPI)_2_ on PC3 xenographs.

**Table S6.** Biodistribution data of 10 pmol [^68^Ga]Ga-DOTAGA.Glu.(FAPI)_2_ and 10 pmol [^68^Ga]Ga-DOTAGA.Glu.(FAPI)_2_ co-injected with 500 pmol DOTAGA.Glu.(FAPI)_2_ to healthy mice. The comparisons were made between these two groups and the concentration of 10 pmol [^68^Ga]Ga-DOTAGA.Glu.(FAPI)_2_ was serving as a reference. Adjusted p values are presented at at the 0.05 level. Data are presented as % IA/g of tissue and are presented as mean ± SD (*n*=3). SD – standard deviation.

| **Organs** | **[^68^Ga]Ga-DOTAGA.Glu.(FAPI)_2_** | **[^68^Ga]Ga-DOTAGA.Glu.(FAPI)_2_ + DOTAGA.Glu.(FAPI)_2_** | | **Adjusted P value (at 0,05 level)** |
| --- | --- | --- | --- | --- |
| blood  heart  gallbladder  liver  spleen  lung  kidney  stomach  intestine  adrenal  pancreas  muscle  bone  salivary glands | 23±2.7  8.4± 2.5  3.1±0.03  4.7±0.5  4.4±0.7  8±0.04  8±0.01  2.2±0.3  2.8±0.07  nd  6.1±0.4  2.5 ±0.4  3.1±1  14.5±5.6 | 3.5±0.4  1.4±0.4  nd  1.1±0.2  0.9±0.2  1.3±0.4  3.6±0.8  1±0.01  1.2± 0.04  2.3±0.2  2.4±0.2  1.9±0.4  3.1±0.5  7.3±0.1 |  | <0.001  <0.001  nd  0.01  0.01  <0.001  <0.001  0.79  0.54  nd  0.009  >0.99  >0.99  <0.001 |

**Table S7.** Biodistribution data of 10 pmol [^68^Ga]Ga-DOTAGA.Glu.(FAPI)_2_ co-injected with various combinations of inhibitors in the mass of 500 pmol to healthy mice. The comparisons were made between injected inhibitor combination and the concentration of 10 pmol of [^68^Ga]Ga-DOTAGA.Glu.(FAPI)_2_ (Tab.S7), serving as a reference. The table should be read column-wise. The same letters indicate the absence of significant difference (at the 0.05 level) between the injected combinations. Data are presented as % IA/g of tissue and are presented as mean ± SD (*n*=3). SD – standard deviation.

| **Organ** | **[^68^Ga]Ga-DOTAGA.Glu.(FAPI)_2_ + DPP4 inhibitor** | **[^68^Ga]Ga-DOTAGA.Glu.(FAPI)_2_ + PREP inhibitor (Salidroside)** | **[^68^Ga]Ga-DOTAGA.Glu.(FAPI)_2_ + PREP inhibitor KYP-2047** | **[^68^Ga]Ga-DOTAGA.Glu.(FAPI)_2_ + PREP inhibitor Baicalin** | **[^68^Ga]Ga-DOTAGA.Glu.(FAPI)_2_ + Salidroside + DPP4 inhibitor** | **[^68^Ga]Ga-DOTAGA.Glu.(FAPI)_2_ + DOTAGA.Glu.(FAPI)_2_** **+ Salidroside + DPP4 inhibitor** |
| --- | --- | --- | --- | --- | --- | --- |
| blood  heart  gallbladder  liver  spleen  lung  kidney  stomach  intestine  adrenal  pancreas  muscle  bone  salivary glands | 21..7±1.1^a^  6.5±0.4^a^  2.1±3.3  5.1±0.3^a^  5.1±0.6^a^  9.1±0.8^a^  7.5±0.4^a^  2.4±0.4^a^  3.4±0.2^a^  4.6±2.8  10.7±1.2^b^  2.4±0.4^a^  3.2±1.4^a^  14.4±1.1^a^ | 18.9±0.2^b^  6.4±0.7^a^  1.4±0.4  5.1±0.6^a^  4.7±0.8^a^  10.6±0.4^a^  7±0.6^a^  2.7±0.4^a^  3.1±0.3^a^  7.7±2.1  11.2±1.4^b^  2.3±0.4^a^  3±2.6^a^  10.5±1.9^b^ | 21.5±0.5^a^  7.7±1.1^a^  1.7±1  4.9±0.09^a^  4.4±0.4^a^  6.9±1.4^a^  6.6±0.3^a^  2.6±0.07^a^  3.3±0.2^a^  9.6±1.4  11.4±1.1^b^  2.5±0.2^a^  3.7±0.6^a^  12.6±2.3^a^ | 22±3.5^a^  7.4±1.1^a^  3.3±1.8  5.3±0.5^a^  5±1.1^a^  9.6±1.2^a^  7.5±1.5^a^  2.2±0.3^a^  3±0.5^a^  9.4±2.5  6.7±0.3^a^  2.2±0.7^a^  3.8±1.28^a^  12.9±3.1^a^ | 20±1.8^b^  6.7±0.3^a^  3.2±1.8  5.3±0.2^a^  5.5±0.2^a^  10.4±0.4^a^  7.5±0.3^a^  3.1±0.2^a^  3.4±0.5^a^  11±3.6  11.3±0.9^b^  2.5±0.2^a^  3.3±0.5^a^  10.5±2.5^b^ | 3.3±0.1^b^  1.3±0.3^b^  1.2±0.6  1.7±0.1^b^  1.6±0.5^b^  2.6±1.8^b^  3.5±0.2^b^  0.9±0.2^a^  0.9±0.2^a^  2.6±1.1  2.1±0.2^b^  2.1±0.2^a^  2.7±0.03^a^  9.04±1.3^b^ |

**Figure S9**. Biodistribution data of 10 pmol [^68^Ga]Ga-DOTAGA.Glu.(FAPI)_2_ co-injected with various combinations of inhibitors with a the mass of 500 pmol in each case to healthy mice. Data are presented as % IA/g of tissue and are presented as mean ± SD (*n*=3). SD – standard deviation.


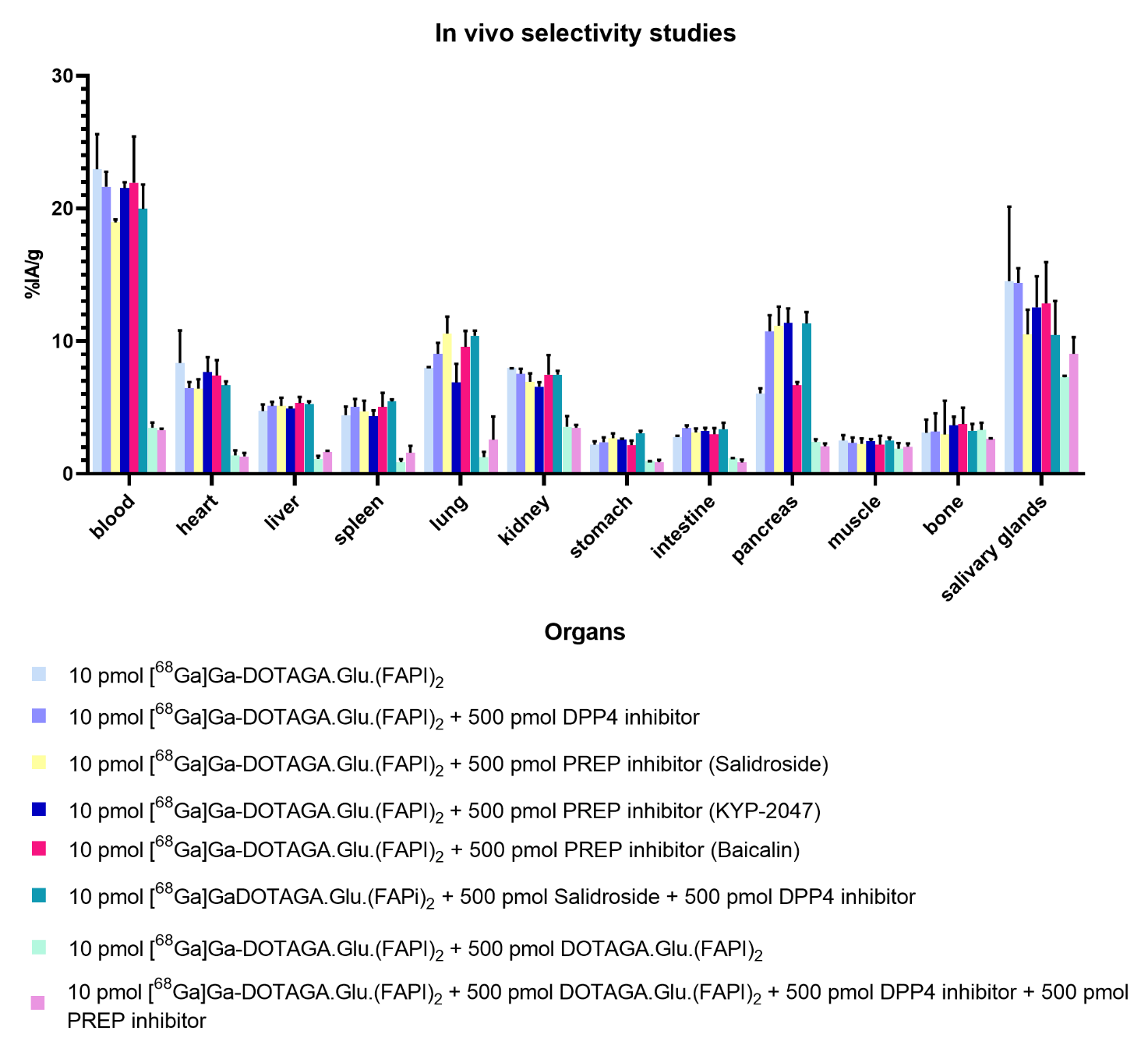


**Table S8.** Biodistribution data of 600 pmol [^68^Ga]Ga-DOTAGA.Glu.(FAPI)_2_ and 600 pmol [^68^Ga]Ga-DOTAGA.Glu.(FAPI)_2_ co-injected with 500 pmol DPP4 and 500 pmol PREP (salidroside) inhibitor to PC3 xenografts. Adjusted p values are presented at at the 0.05 level. Data are presented as % IA/g of tissue and are presented as mean ± SD (*n*=3). SD – standard deviation.

| **Organs** | **Injected inhibitor combination to PC3 xenografts** | | |
| --- | --- | --- | --- |
|  | **[^68^Ga]Ga-DOTAGA.Glu.(FAPI)_2_** | **[^68^Ga]Ga-DOTAGA.Glu.(FAPI)_2_ + Salidroside + DPP4 inhibitor** | **Adjusted P value (at 0,05 level)** |
| blood  heart  liver  spleen  lung  kidney  stomach  intestine  pancreas  muscle  bone  tumor  salivary glands | 3.2±0.5  1.4±0.1  2.1±0.2  1.3±0.3  2.7±0.3  6.5±1.1  1.5±0.08  1.4±0.2  2.7±0.8  1.6±0.2  4.9±1.3  16.5±2.3  8.2±0.5 | 3.9±0.4  1.5±0.1  2.4±0.6  2.3±0.2  4.2±2.6  6.4±1.9  1.1±0.05  1.3±0.4  2.4±0.2  2.1±0.2  7±3.1  19.4±1.6  8.4±1.6 | >0.9999  >0.9999  >0.9999  0.9982  >0.9999  >0.9999  >0.9999  >0.9999  >0.9999  >0.9999  0.6823  0.2300  >0.9999 |

**Table S9.** Primer sequences for the *GAPDH, RLPL0* and *FAP* genes used in qPCR. Primers were designed based on mouse genome sequence: GRCm39 (*FAP* – GenBank ID:14089).

| **Gene** | **Primer sequence** | **Product size (bp)** | **Annealing temperature (ºC)** |
| --- | --- | --- | --- |
| ***GAPDH*** | F: ATGTGTCCGTCGTGGATCTGA  R: ATGCCTGCTTCACCACCTTCT | 77 | 60 |
| ***RLPL0*** | F: GGACCCGAGAAGACCTCCTT  R: GCACATCACTCAGAATTTCAATGG | 85 | 60 |
| ***FAP_M*** | F: GGATGGGCTGGTGGATTCTT  R: CCTCCCACTTGCCACTTGTA | 157 | 60 |

**Table. S10.** The p-values for selected critical organs, calculated within a 95% confidence interval for the following radiopharmaceuticals: [^68^Ga]Ga-DOTA.SA.FAPI, [^68^Ga]Ga-DATA^5m^.SA.FAPI, [^68^Ga]Ga-DO3A.Glu.(FAPI)_2_, [^68^Ga]Ga-DOTAGA.Glu.(FAPI)_2_, and [^68^Ga]Ga-DOTAGA.(SA.FAPI)_2_. The comparisons were made between the total injected mass of each radiopharmaceutical and a reference mass of 10 pmol.

| **[^68^Ga]Ga-DOTA.SA.FAPI** | | | | |
| --- | --- | --- | --- | --- |
|  | **Blood** | **Pancreas** | **Bone** | **Tumor** |
| **100 pmol vs. 10 pmol** | <0.001 | <0.001 | >0.99 | <0.001 |
| **350 pmol vs. 10 pmol** | <0.001 | <0.001 | 0.003 | <0.001 |
| **600 pmol vs. 10 pmol** | <0.001 | <0.001 | >0.99 | <0.001 |
| **1000 pmol vs. 10 pmol** | <0.001 | <0.001 | 0.03 | 0.09 |
| **1500 pmol vs. 10 pmol** | <0.001 | <0.001 | 0.01 | 0.01 |
| **[^68^Ga]Ga-DATA^5m^.SA.FAPI** | | | | |
|  | **Blood** | **Pancreas** | **Bone** | **Tumor** |
| **100 pmol vs. 10 pmol** | 0.19 | 0.77 | >0.99 | 0.003 |
| **350 pmol vs. 10 pmol** | 0.002 | 0.006 | >0.99 | <0.001 |
| **600 pmol vs. 10 pmol** | <0.001 | 0.002 | >0.99 | 0.01 |
| **1000 pmol vs. 10 pmol** | <0.001 | 0.001 | 0.93 | 0.12 |
| **1500 pmol vs. 10 pmol** | <0.001 | <0.001 | 0.86 | 0.91 |
| **[^68^Ga]Ga-DO3A.Glu.(FAPI)_2_** | | | | |
|  | **Blood** | **Pancreas** | **Bone** | **Tumor** |
| **100 pmol vs. 10 pmol** | <0.001 | <0.001 | <0.001 | 0.30 |
| **350 pmol vs. 10 pmol** | <0.001 | <0.001 | <0.001 | <0.001 |
| **600 pmol vs. 10 pmol** | <0.001 | <0.001 | <0.001 | <0.001 |
| **1000 pmol vs. 10 pmol** | <0.001 | <0.001 | <0.001 | <0.001 |
| **1500 pmol vs. 10 pmol** | <0.001 | <0.001 | <0.001 | 0.15 |
| **[^68^Ga]Ga-DOTAGA.Glu.(FAPI)_2_** | | | | |
|  | **Blood** | **Pancreas** | **Bone** | **Tumor** |
| **100 pmol vs. 10 pmol** | <0.001 | <0.001 | 0.01 | <0.001 |
| **350 pmol vs. 10 pmol** | <0.001 | <0.001 | 0.35 | 0.02 |
| **600 pmol vs. 10 pmol** | <0.001 | <0.001 | >0.99 | <0.001 |
| **1000 pmol vs. 10 pmol** | <0.001 | <0.001 | 0.02 | 0.005 |
| **1500 pmol vs. 10 pmol** | <0.001 | <0.001 | 0.04 | <0.001 |
| **[^68^Ga]Ga-DOTAGA.(SA.FAPI)_2_** | | | | |
|  | **Blood** | **Pancreas** | **Bone** | **Tumor** |
| **100 pmol vs. 10 pmol** | <0.001 | 0.01 | 0.72 | 0.35 |
| **350 pmol vs. 10 pmol** | <0.001 | <0.001 | >0.99 | <0.001 |
| **600 pmol vs. 10 pmol** | <0.001 | <0.001 | >0.99 | <0.001 |
| **1000 pmol vs. 10 pmol** | <0.001 | <0.001 | >0.99 | <0.001 |
| **1500 pmol vs. 10 pmol** | <0.001 | <0.001 | 0.67 | <0.001 |
